# Correction: Low-dose calcium supplementation during pregnancy in low and middle-income countries: A cost-effectiveness analysis

**DOI:** 10.1371/journal.pgph.0005647

**Published:** 2025-12-15

**Authors:** The PLOS One Staff

## Notice of Republication

This article was republished on December 2, 2025 to correct an error in which author Pratibha Dwarkanath was not listed as a co-first author alongside Happiness P. Saronga and Hening Cui.

The publisher apologizes for these errors. The originally published, uncorrected article and the republished, corrected articles are provided here for reference.

## Supporting information

S1 File Originally published, uncorrected article. (PDF)

S2 File Republished, corrected article. (PDF)
