# Supplementary material for: Low-dose calcium supplementation during pregnancy in low and middle-income countries: A cost-effectiveness analysis
Source: PLOS Glob Public Health. 2025 Sep 2;5(9):e0004002. doi: 10.1371/journal.pgph.0004002 (PMC12404394; doi:10.1371/journal.pgph.0004002)
Supplement: S1 File — Table A: Data inputs definitions, values, and sources. Fig A: Comparison of modelled estimates of deaths from preterm birth and preeclampsia to Global Burden of Disease Study estimates for each country. Fig B: Comparison of modelled estimates of DALYs from preterm birth and preeclampsia to Global Burden of Disease Study estimates for each country. Fig C: Availability of analytic inputs for each country. Table B: Deaths and DALYs averted by low-dose calcium supplementation intervention in 2024, by condition, age group, form of health loss. Table C: Health effects estimated for low-dose calcium supplementation intervention in 2024 for each low- and middle-income country, compared to no intervention. Table D: Intervention costs, cost savings, and cost-effectiveness of the low-dose calcium supplementation intervention in 2024 for each low- and middle-income country, compared to no intervention. Table E: Results of one-way sensitivity analyses for each parameter, for the incremental cost-effectiveness ratio (ICER) and net monetary benefit (NMB) across all low- and middle-income countries. Table F: Health impact results with alternative analytic assumptions. Table G: Cost and cost-effectiveness results with alternative analytic assumptions. Fig D: Country-specific health benefits and intervention costs (excluding cost-savings) compared to cost-effectiveness thresholds for each country. (PDF) [file pgph.0004002.s001.pdf]

S1 File for "Low-dose calcium supplementation during pregnancy in low and middle-income countries: a cost-effectiveness analysis".

Contents

Contents	Page(s)
**Table A**: Data inputs definitions, values, and sources	2
**Fig A**: Availability of analytic inputs for each country	3
**Table B**: Deaths and DALYs averted by low-dose calcium supplementation intervention in 2024, by condition, age group, form of health loss	4
**Table C**: Health effects estimated for low-dose calcium supplementation intervention in 2024 for each low- and middle-income country, compared to no intervention	5-8
**Table D**: Intervention costs, cost savings, and cost-effectiveness of the low-dose calcium supplementation intervention in 2024 for each low- and middle-income country, compared to no intervention	9-13
**Table E**: Results of one-way sensitivity analyses for each parameter, for the incremental cost-effectiveness ratio (ICER) and net monetary benefit (NMB) across all low- and middle-income countries	14
**Table F**: Health impact results with alternative analytic assumptions	15
**Table G**: Cost and cost-effectiveness results with alternative analytic assumptions	16
**Fig B**: Country-specific health benefits and intervention costs (excluding cost-savings) compared to cost-effectiveness thresholds for each country	17
**Fig C-1**: Country-specific input values (ANC coverage)	18
**Fig C-2**: Country-specific input values (number of livebirths)	18
**Fig C-3**: Country-specific input values (proportion of births that are preterm)	19
**Fig C-4**: Country-specific input values (proportion of births with preeclampsia)	19
**Fig C-5**: Country-specific input values (intervention adherence)	20
**Fig C-6**: Country-specific input values (infant deaths per preterm birth)	20
**Fig C-7**: Country-specific input values (maternal deaths per preeclampsia case)	21
**Fig C-8**: Country-specific input values (infant YLLs per preterm birth)	21
**Fig C-9**: Country-specific input values (maternal YLLs per preeclampsia case)	22
**Fig C-10**: Country-specific input values (infant YLDs per preterm birth)	22
**Fig C-11**: Country-specific input values (maternal YLDs per preterm birth)	23
**Fig C-12**: Country-specific input values (maternal YLDs per preeclampsia case)	23
**Fig C-13**: Country-specific input values (unit cost for preterm birth without death)	24
**Fig C-14**: Country-specific input values (unit cost for preterm birth with death)	24
**Fig C-15**: Country-specific input values (unit cost for preeclampsia)	25
**Fig C-16**: Country-specific input values (willingness to pay threshold)	25
**Table H**: Methods used for imputation and data processing for country-level inputs	26-28
Equations used to calculate each study outcome	29-31
Citations	32-33

Data input	Notation	Value (95% interval)	Probability distribution	Source
Indicator for whether a country (i) is classified as low dietary calcium (1 for low-calcium settings, 0 otherwise).	a_i	125 of 129 countries identified as having low dietary calcium	No uncertainty considered	1,2
ANC coverage in country i	b_i	Country-specific (Fig C-1)	Beta distribution	3
Number of livebirths for 2024 in country i	c_i	Country-specific (Fig C-2)	Gamma distribution	4
Proportion of births that are preterm in country i	d_i	Country-specific (Fig C-3)	Beta distribution	5
Proportion of births with preeclampsia in country i	e_i	Country-specific (Fig C-4)	Beta distribution	6,7
Risk ratio of preterm birth with vs. without calcium supplementation	f	0.76 (0.60, 0.97)	Gamma distribution	8
Risk ratio of preeclampsia with vs. without calcium supplementation	g	0.45 (0.31, 0.65)	Gamma distribution	8
Intervention adherence in country i	h_i	Country-specific (Fig C-5)	Beta distribution	9
Reduction in intervention impact for low adherence	j	100% (50% examined in sensitivity analyses)	No uncertainty considered	Assumption
Infant deaths per preterm birth in country i	$m_i^{inf\_ptb}$	Country-specific (Fig C-6)	Beta distribution	6
Maternal deaths per preeclampsia case in country i	$m_i^{mat\_pe}$	Country-specific (Fig C-7)	Beta distribution	6,7
Infant YLLs (Years of Life Lost) per preterm birth in country i	$n_i^{inf\_ptb}$	Country-specific (Fig C-8)	Gamma distribution	6
Maternal YLLs per preeclampsia case in country i	$n_i^{mat\_pe}$	Country-specific (Fig C-9)	Gamma distribution	6,7
Infant YLDs (Years Lived with Disability) per preterm birth in country i	$o_i^{inf\_ptb}$	Country-specific (Fig C-10)	Gamma distribution	6
Maternal YLDs per preterm birth in country i	$o_i^{mat\_ptb}$	Country-specific (Fig C-11)	Gamma distribution	6
Maternal YLDs per preeclampsia case in country i	$o_i^{mat\_pe}$	Country-specific (Fig C-12)	Gamma distribution	6,7
Unit cost of additional health services per preterm birth without death, as compared to a full term delivery, in country i	p_i^{ptb1}	Country-specific (Fig C-13)	Normal distribution for log cost	10-12
Unit cost of additional health services per preterm birth with death, as compared to a full term delivery, in country i	p_i^{ptb2}	Country-specific (Fig C-14)	Normal distribution for log cost	10-12
Unit cost of additional health services per pre-eclampsia episode, as compared to no eclampsia, in country i	p_i^{pe}	Country-specific (see Fig C-15)	Normal distribution for log cost	10,12
Unit cost per calcium tablet	p^{calc}	US$0.02 ($0.015 examined in sensitivity analyses)	No uncertainty considered	13
Mark-up for supply chain	q^{sc}	13% (5, 20)	Beta distribution	14
Wastage rate	q^{waste}	6% (3, 10)	Beta distribution	15
Willingness to pay threshold, for country i	λ_i	Country-specific (Fig C-16)	Uniform distribution	16

Table A: Data inputs definitions, values, and sources.

ANC = antenatal care, YLLs = Years of Life Lost, YLDs = Years Lived with Disability. US$ = 2022 US dollars.

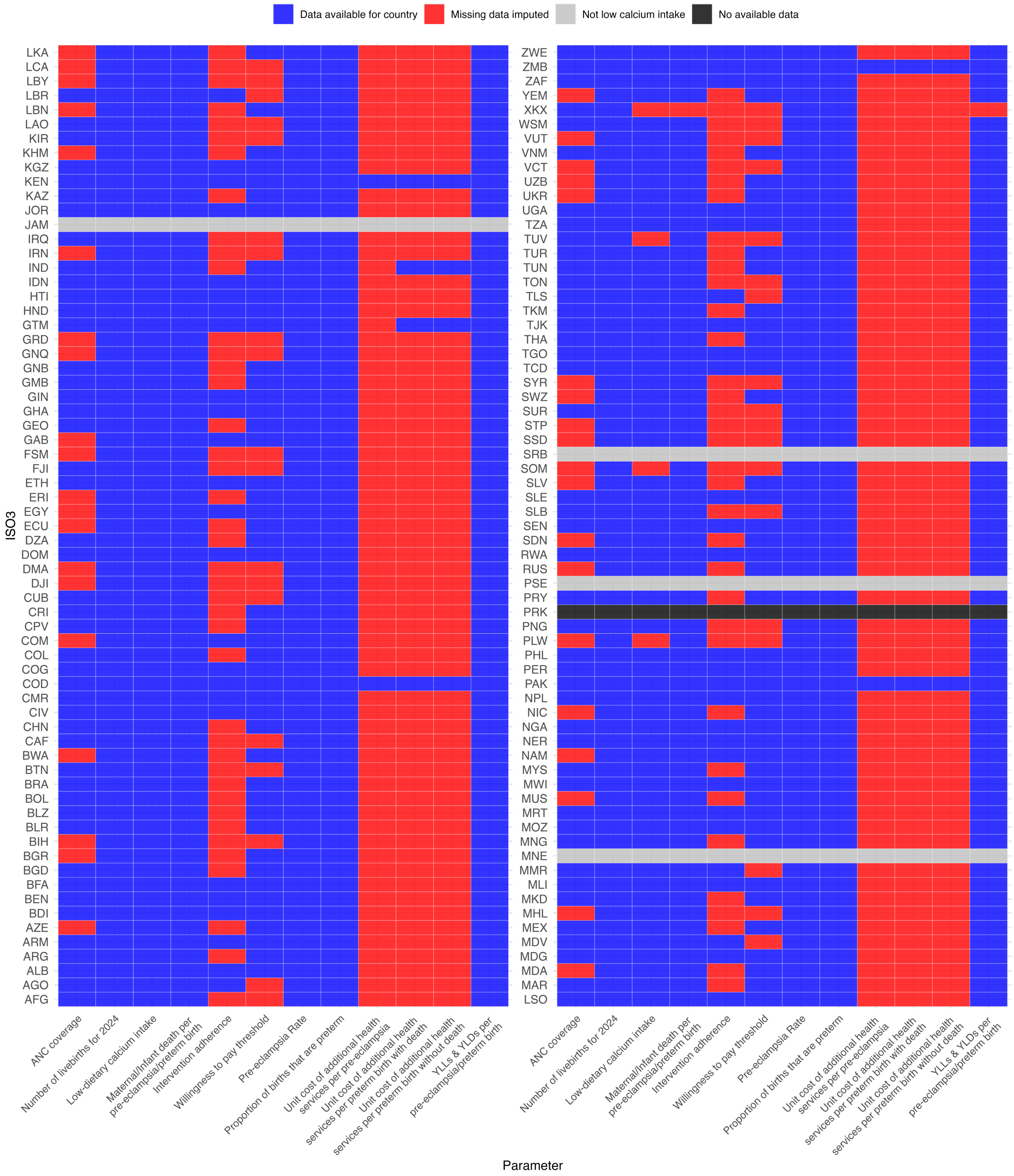

Fig A: Availability of analytic inputs for each country.

Table H describes the approach to imputation and data processing for each country-level input.

Country grouping	Deaths averted by age group		DALYs averted by condition		DALYs averted by age group		DALYs averted by form of health loss	
	Infant deaths averted (thousands)	Maternal deaths averted (thousands)	DALYs due to preterm birth averted (thousands)	DALYs due to preeclampsia averted (thousands)	DALYs averted among infants (thousands)	DALYs averted among mothers (thousands)	DALYs due to premature death averted (thousands)	DALYs due nonfatal health losses averted (thousands)
All LMIC	57.7 (9.2, 128.4)	7.2 (3.7, 12.4)	5455 (893, 12347)	476 (246, 804)	5233 (854, 11914)	697 (370, 1134)	5554 (1194, 12159)	377 (98, 749)
World Region								
Africa	19.5 (2.8, 47.1)	2.3 (1.1, 4.1)	1776 (253, 4355)	155 (77, 267)	1753 (249, 4308)	178 (92, 300)	1866 (367, 4415)	65 (21, 133)
Americas	4.1 (0.7, 8.1)	0.7 (0.4, 1.1)	404 (71, 796)	45 (24, 73)	376 (66, 741)	73 (40, 113)	407 (102, 768)	42 (11, 78)
Eastern Mediterranean	8.7 (1.2, 21.2)	1.8 (0.9, 3.2)	803 (114, 1967)	113 (55, 199)	783 (111, 1925)	133 (67, 228)	876 (201, 2027)	40 (9, 88)
Europe	1.7 (0.3, 3.4)	0.1 (0.1, 0.2)	174 (30, 344)	8 (4, 15)	159 (27, 317)	23 (10, 39)	159 (32, 314)	23 (6, 42)
Southeast Asia	18.7 (3.1, 38.9)	1.9 (1.0, 3.2)	1787 (303, 3787)	126 (68, 206)	1700 (287, 3603)	214 (111, 348)	1776 (382, 3658)	137 (30, 280)
Western Pacific	5.0 (0.8, 10.7)	0.4 (0.2, 0.6)	510 (87, 1079)	28 (15, 46)	462 (78, 988)	76 (33, 131)	469 (95, 985)	70 (17, 134)
Income Level								
Low income	9.1 (1.2, 23.4)	1.3 (0.6, 2.3)	830 (109, 2138)	82 (37, 150)	821 (108, 2113)	91 (43, 165)	883 (168, 2179)	28 (8, 62)
Lower middle-income	36.9 (5.9, 82.7)	4.5 (2.3, 7.8)	3456 (560, 7859)	296 (153, 501)	3340 (539, 7630)	412 (218, 674)	3547 (754, 7809)	205 (52, 421)
Upper middle-income	11.7 (2.0, 23.7)	1.4 (0.8, 2.3)	1169 (204, 2358)	98 (53, 158)	1072 (188, 2181)	195 (99, 313)	1123 (257, 2216)	143 (35, 269)

Table B: Deaths and DALYs averted by low-dose calcium supplementation intervention in 2024, by condition, age group, form of health loss.

DALY = disability-adjusted life year. LMIC = low- and middle-income countries. Values in parentheses represent equal-tailed 95% uncertainty intervals.

Country	Preterm births averted (thousands)	Preterm births averted (% of base-case)	Preeclampsia cases averted (thousands)	Preeclampsia cases (% of base-case)	Infant and maternal deaths averted	DALYs averted (thousands)
Afghanistan	7 (0.08, 29)	3 (1, 7)	4 (2, 9)	8 (3, 14)	251 (32, 937)	21 (2, 81)
Angola	13 (2, 30)	10 (2, 18)	28 (15, 43)	23 (14, 31)	849 (202, 1849)	74 (16, 168)
Albania	0.3 (0.05, 1)	14 (2, 24)	0.3 (0.1, 0.4)	32 (20, 42)	7 (1, 14)	0.7 (0.1, 1)
Argentina	9 (2, 17)	17 (3, 30)	12 (6, 19)	39 (25, 52)	531 (109, 989)	51 (10, 96)
Armenia	0.4 (0.07, 1)	17 (3, 30)	0.4 (0.2, 1)	39 (25, 53)	10 (2, 18)	1 (0.2, 2)
Azerbaijan	2 (0.3, 4)	16 (3, 29)	2 (1, 3)	37 (23, 50)	120 (20, 273)	11 (2, 26)
Burundi	2 (0.3, 6)	6 (1, 11)	5 (2, 9)	13 (6, 21)	121 (25, 300)	11 (2, 27)
Benin	3 (0.4, 7)	8 (1, 14)	9 (5, 13)	18 (11, 25)	264 (47, 660)	23 (4, 59)
Burkina Faso	5 (1, 12)	7 (1, 12)	11 (6, 18)	15 (9, 22)	251 (57, 579)	22 (5, 52)
Bangladesh	29 (5, 60)	6 (1, 11)	21 (11, 34)	14 (8, 19)	629 (193, 1294)	61 (16, 131)
Bulgaria	0.7 (0.1, 1)	17 (3, 30)	0.6 (0.3, 0.9)	39 (24, 52)	15 (3, 29)	2 (0.3, 3)
Bosnia and Herzegovina	0.3 (0.05, 1)	16 (3, 29)	0.3 (0.2, 1)	37 (23, 50)	7 (1, 14)	1 (0.2, 2)
Belarus	1 (0.2, 2)	18 (3, 32)	2 (1, 3)	41 (26, 56)	9 (2, 17)	1 (0.3, 3)
Belize	0.1 (0.02, 0.2)	17 (3, 29)	0.2 (0.09, 0.3)	38 (24, 51)	4 (1, 7)	0.3 (0.08, 1)
Bolivia	3 (1, 7)	14 (3, 25)	3 (1, 5)	33 (21, 44)	287 (87, 575)	24 (6, 51)
Brazil	50 (9, 90)	17 (3, 30)	49 (29, 74)	39 (25, 52)	1707 (429, 3050)	166 (38, 299)
Bhutan	0.2 (0.003, 1)	14 (3, 25)	0.2 (0.08, 0.3)	32 (20, 44)	10 (1, 36)	1 (0.08, 3)
Botswana	1 (0.06, 2)	10 (2, 21)	1 (1, 2)	24 (11, 37)	28 (3, 86)	3 (0.3, 8)
Central African Republic	1 (0.2, 3)	5 (1, 10)	3 (1, 4)	12 (6, 18)	120 (25, 287)	10 (2, 25)
China	114 (20, 212)	17 (3, 30)	124 (71, 189)	40 (26, 53)	2737 (541, 5116)	292 (57, 546)
Côte d'Ivoire	10 (1, 24)	8 (1, 15)	17 (10, 26)	19 (12, 26)	603 (116, 1437)	53 (10, 131)
Cameroon	9 (1, 21)	10 (2, 18)	21 (12, 33)	23 (14, 31)	769 (175, 1713)	67 (15, 155)
Congo, Dem. Rep.	31 (5, 74)	6 (1, 11)	49 (26, 81)	13 (7, 20)	2237 (461, 5235)	197 (37, 471)
Congo, Rep.	2 (0.3, 5)	13 (2, 22)	5 (3, 7)	29 (18, 40)	129 (38, 274)	11 (3, 25)
Colombia	10 (2, 19)	16 (3, 28)	10 (6, 15)	37 (23, 49)	254 (76, 477)	24 (6, 46)
Comoros	0.2 (0.03, 1)	9 (1, 18)	0.5 (0.2, 1)	20 (8, 34)	8 (2, 21)	1 (0.1, 2)
Cabo Verde	0.1 (0.02, 0.3)	14 (3, 25)	0.3 (0.2, 0.5)	33 (21, 45)	5 (1, 11)	0.5 (0.08, 1)
Costa Rica	1 (0.2, 2)	18 (3, 31)	1 (1, 2)	40 (26, 54)	26 (6, 54)	3 (1, 6)
Cuba	1 (0.2, 2)	15 (3, 25)	2 (1, 3)	33 (21, 44)	9 (3, 15)	1 (0.4, 2)
Djibouti	0.3 (0.04, 1)	13 (2, 23)	0.7 (0.3, 1)	29 (16, 42)	15 (4, 34)	1 (0.3, 3)
Dominica	0.01 (0.002, 0.03)	16 (3, 28)	0.02 (0.01, 0.03)	37 (23, 50)	0.5 (0.09, 1)	0.05 (0.009, 0.1)
Dominican Republic	3 (0.5, 6)	17 (3, 30)	5 (3, 7)	39 (25, 52)	160 (48, 315)	14 (4, 29)
Algeria	9 (2, 21)	12 (2, 21)	13 (7, 21)	27 (17, 37)	879 (159, 1971)	81 (14, 186)
Ecuador	3 (0.5, 8)	16 (3, 28)	9 (6, 14)	36 (23, 49)	190 (49, 433)	17 (4, 40)

Country	Preterm births averted (thousands)	Preterm births averted (% of base-case)	Preeclampsia cases averted (thousands)	Preeclampsia cases (% of base-case)	Infant and maternal deaths averted	DALYs averted (thousands)
Egypt, Arab Rep.	28 (4, 65)	13 (2, 24)	39 (20, 63)	30 (17, 42)	191 (63, 422)	22 (6, 49)
Eritrea	1 (0.09, 2)	8 (1, 17)	2 (1, 3)	18 (6, 32)	33 (8, 83)	3 (1, 7)
Ethiopia	33 (5, 76)	6 (1, 11)	50 (28, 78)	14 (8, 20)	1228 (266, 2775)	111 (23, 256)
Fiji	0.2 (0.03, 0.5)	15 (3, 27)	0.3 (0.2, 1)	35 (23, 47)	12 (2, 26)	1 (0.2, 2)
Micronesia, Fed. Sts.	0.02 (0.003, 0.06)	13 (2, 25)	0.04 (0.02, 0.07)	31 (15, 45)	1 (0.2, 2)	0.07 (0.02, 0.2)
Gabon	0.5 (0.06, 1)	10 (2, 21)	1 (1, 3)	24 (11, 38)	25 (6, 64)	2 (0.5, 6)
Georgia	0.5 (0.1, 1)	15 (3, 26)	0.3 (0.2, 0.5)	35 (22, 46)	12 (3, 21)	1 (0.3, 2)
Ghana	10 (2, 23)	13 (2, 24)	14 (9, 22)	31 (19, 42)	415 (89, 979)	38 (8, 92)
Guinea	2 (0.4, 6)	5 (1, 10)	5 (3, 8)	12 (7, 18)	188 (47, 422)	16 (4, 37)
Gambia, The	1 (0.1, 2)	11 (2, 20)	2 (1, 3)	25 (15, 36)	47 (13, 108)	4 (1, 10)
Guinea-Bissau	1 (0.1, 2)	11 (2, 20)	2 (0.8, 2)	25 (15, 36)	42 (10, 95)	4 (1, 9)
Equatorial Guinea	0.5 (0.07, 1)	10 (2, 20)	1 (0.4, 2)	23 (11, 37)	26 (5, 65)	2 (0.4, 6)
Grenada	0.03 (0.005, 0.06)	16 (3, 29)	0.06 (0.03, 0.09)	37 (24, 50)	1 (0.4, 2)	0.1 (0.03, 0.2)
Guatemala	6 (1, 15)	15 (3, 26)	3 (2, 4)	34 (22, 46)	192 (71, 387)	16 (5, 35)
Honduras	3 (0.5, 6)	14 (3, 26)	3 (1, 4)	33 (21, 45)	113 (38, 221)	10 (3, 20)
Haiti	2 (0.4, 5)	10 (2, 18)	3 (1, 5)	23 (14, 32)	115 (49, 214)	9 (3, 17)
Indonesia	43 (7, 95)	13 (2, 23)	82 (48, 122)	31 (19, 41)	3050 (930, 6168)	266 (69, 559)
India	284 (48, 575)	9 (2, 17)	299 (170, 456)	21 (13, 29)	16161 (3466, 32063)	1514 (306, 3084)
Iran, Islamic Rep.	11 (2, 25)	13 (2, 24)	16 (8, 27)	30 (17, 43)	405 (67, 919)	40 (7, 92)
Iraq	13 (2, 29)	12 (2, 21)	15 (8, 25)	27 (17, 37)	538 (99, 1211)	50 (9, 116)
Jordan	3 (1, 7)	16 (3, 27)	2 (1, 4)	36 (23, 48)	105 (20, 238)	10 (2, 23)
Kazakhstan	4 (1, 7)	18 (3, 31)	8 (4, 12)	41 (26, 54)	140 (29, 271)	13 (3, 26)
Kenya	12 (2, 27)	9 (2, 16)	29 (16, 44)	21 (13, 29)	485 (152, 969)	44 (12, 91)
Kyrgyz Republic	2 (0.3, 3)	14 (3, 26)	2 (0.8, 2)	33 (20, 45)	107 (25, 202)	10 (2, 19)
Cambodia	2 (0.3, 7)	12 (2, 24)	6 (2, 10)	28 (13, 43)	149 (28, 386)	13 (2, 35)
Kiribati	0.03 (0.004, 0.06)	10 (2, 18)	0.04 (0.02, 0.06)	23 (14, 33)	2 (0.4, 5)	0.2 (0.03, 0.5)
Lao PDR	1 (0.1, 4)	10 (2, 17)	2 (1, 4)	22 (14, 31)	139 (15, 407)	13 (1, 36)
Lebanon	1 (0.1, 2)	13 (2, 24)	0.8 (0.4, 1)	30 (17, 42)	21 (3, 56)	2 (0.3, 6)
Liberia	2 (0.3, 5)	12 (2, 22)	4 (2, 7)	27 (16, 39)	96 (30, 206)	8 (2, 18)
Libya	1 (0.1, 4)	14 (2, 25)	3 (1, 5)	31 (18, 44)	33 (6, 90)	3 (1, 10)
St. Lucia	0.03 (0.005, 0.06)	17 (3, 29)	0.05 (0.02, 0.08)	38 (24, 51)	1 (0.3, 3)	0.1 (0.02, 0.3)
Sri Lanka	2 (0.1, 6)	8 (1, 20)	4 (1, 8)	19 (3, 38)	41 (4, 125)	4 (0.3, 13)
Lesotho	0.6 (0.1, 1)	11 (2, 20)	1 (1, 2)	25 (15, 36)	53 (13, 119)	5 (1, 11)
Morocco	5 (1, 11)	9 (2, 16)	6 (3, 10)	20 (13, 28)	228 (56, 496)	21 (5, 47)

Country	Preterm births averted (thousands)	Preterm births averted (% of base-case)	Preeclampsia cases averted (thousands)	Preeclampsia cases (% of base-case)	Infant and maternal deaths averted	DALYs averted (thousands)
Moldova	0.4 (0.06, 1)	16 (3, 28)	0.9 (0.5, 1)	36 (22, 49)	6 (1, 12)	0.7 (0.2, 1)
Madagascar	6 (1, 14)	6 (1, 12)	12 (6, 21)	15 (8, 22)	269 (75, 606)	23 (6, 54)
Maldives	0.1 (0.02, 0.2)	15 (3, 26)	0.1 (0.08, 0.2)	35 (22, 46)	3 (1, 6)	0.3 (0.06, 1)
Mexico	23 (4, 43)	17 (3, 31)	63 (38, 93)	40 (26, 53)	882 (227, 1634)	84 (20, 157)
Marshall Islands	0.008 (0.001, 0.02)	14 (2, 26)	0.01 (0.005, 0.02)	32 (17, 47)	0.5 (0.1, 1)	0.04 (0.009, 0.09)
North Macedonia	0.2 (0.04, 0.4)	17 (3, 30)	0.3 (0.1, 0.4)	39 (25, 52)	12 (2, 22)	1 (0.2, 2)
Mali	4 (0.5, 10)	6 (1, 11)	12 (6, 19)	14 (8, 20)	254 (46, 651)	22 (4, 58)
Myanmar	6 (1, 13)	8 (1, 15)	12 (6, 19)	19 (12, 28)	470 (113, 1061)	41 (9, 96)
Mongolia	1 (0.08, 2)	15 (3, 27)	0.7 (0.4, 1)	35 (22, 47)	25 (4, 62)	2 (0.4, 6)
Mozambique	6 (1, 14)	7 (1, 13)	16 (8, 27)	15 (8, 23)	394 (99, 891)	34 (8, 79)
Mauritania	2 (0.2, 4)	10 (2, 18)	4 (2, 6)	23 (14, 31)	95 (24, 210)	8 (2, 19)
Mauritius	0.1 (0.02, 0.3)	11 (2, 21)	0.2 (0.1, 0.4)	24 (11, 38)	5 (1, 13)	0.5 (0.09, 1)
Malawi	7 (1, 16)	7 (1, 13)	11 (6, 18)	15 (9, 23)	268 (65, 606)	24 (5, 55)
Malaysia	7 (1, 15)	18 (3, 32)	16 (9, 26)	42 (27, 55)	86 (24, 178)	10 (2, 21)
Namibia	1 (0.09, 2)	10 (2, 20)	1 (1, 2)	23 (10, 37)	21 (4, 52)	2 (0.4, 5)
Niger	6 (1, 14)	5 (1, 9)	11 (6, 19)	11 (6, 17)	223 (50, 521)	20 (4, 46)
Nigeria	75 (11, 180)	9 (2, 16)	165 (96, 247)	21 (13, 28)	6683 (1149, 15757)	595 (101, 1428)
Nicaragua	1 (0.2, 2)	14 (2, 25)	2 (1, 3)	32 (19, 45)	32 (10, 59)	3 (1, 6)
Nepal	8 (1, 18)	12 (2, 21)	7 (4, 12)	26 (16, 37)	128 (53, 242)	12 (4, 24)
Pakistan	76 (12, 174)	8 (1, 14)	70 (38, 111)	18 (11, 26)	5730 (1774, 11817)	482 (128, 1045)
Peru	7 (1, 13)	17 (3, 30)	4 (2, 6)	39 (25, 53)	209 (75, 385)	18 (6, 35)
Philippines	29 (4, 70)	14 (3, 25)	59 (34, 89)	33 (21, 45)	1362 (348, 3079)	120 (27, 283)
Palau	0.003 (0.0004, 0.006)	15 (3, 27)	0.005 (0.002, 0.009)	34 (18, 48)	0.08 (0.01, 0.2)	0.008 (0.001, 0.02)
Papua New Guinea	2 (0.3, 4)	8 (1, 14)	2 (1, 4)	18 (12, 25)	136 (31, 315)	12 (2, 28)
Paraguay	2 (0.3, 3)	15 (3, 27)	2 (1, 4)	35 (22, 46)	51 (16, 96)	5 (1, 9)
Russian Federation	14 (2, 25)	17 (3, 30)	33 (17, 53)	39 (24, 52)	108 (24, 201)	15 (4, 27)
Rwanda	3 (0.2, 8)	7 (1, 12)	6 (3, 9)	15 (9, 22)	184 (43, 477)	16 (3, 43)
Sudan	12 (2, 31)	8 (1, 18)	11 (4, 22)	19 (7, 34)	2042 (329, 5218)	179 (27, 463)
Senegal	5 (1, 11)	8 (1, 15)	10 (6, 16)	19 (12, 28)	233 (57, 520)	21 (5, 47)
Solomon Islands	0.2 (0.03, 0.4)	11 (2, 19)	0.2 (0.1, 0.4)	25 (16, 34)	8 (3, 16)	0.6 (0.2, 1)
Sierra Leone	3 (0.4, 6)	10 (2, 19)	5 (3, 9)	23 (13, 34)	210 (56, 475)	18 (4, 41)
El Salvador	1 (0.2, 2)	15 (3, 27)	2 (0.8, 2)	35 (22, 48)	35 (9, 68)	3 (1, 7)
Somalia	6 (1, 15)	7 (1, 17)	12 (4, 24)	17 (6, 32)	268 (44, 722)	23 (4, 64)
South Sudan	3 (0.3, 7)	8 (1, 18)	6 (2, 11)	19 (7, 34)	156 (23, 417)	14 (2, 37)

Country	Preterm births averted (thousands)	Preterm births averted (% of base-case)	Preeclampsia cases averted (thousands)	Preeclampsia cases (% of base-case)	Infant and maternal deaths averted	DALYs averted (thousands)
São Tomé and Príncipe	0.06 (0.008, 0.1)	9 (1, 19)	0.1 (0.06, 0.2)	21 (9, 35)	1 (0.3, 3)	0.1 (0.03, 0.3)
Suriname	0.2 (0.03, 0.4)	12 (2, 21)	0.2 (0.1, 0.3)	27 (17, 36)	10 (2, 23)	1 (0.2, 2)
Eswatini	0.3 (0.04, 1)	10 (2, 20)	0.6 (0.2, 1)	22 (10, 36)	16 (3, 40)	1 (0.2, 4)
Syrian Arab Republic	5 (1, 12)	10 (2, 20)	7 (3, 13)	23 (11, 37)	101 (16, 261)	11 (2, 27)
Chad	3 (0.5, 8)	4 (1, 8)	7 (3, 12)	10 (5, 15)	275 (54, 671)	24 (5, 59)
Togo	2 (0.3, 5)	8 (1, 14)	5 (2, 7)	18 (10, 26)	121 (24, 285)	11 (2, 26)
Thailand	7 (1, 16)	16 (3, 28)	18 (10, 28)	37 (24, 49)	110 (30, 227)	13 (3, 27)
Tajikistan	2 (0.3, 3)	9 (2, 17)	2 (1, 3)	21 (13, 30)	55 (11, 115)	5 (1, 11)
Turkmenistan	2 (0.3, 3)	17 (3, 31)	1 (1, 2)	40 (26, 54)	147 (30, 289)	13 (3, 26)
Timor-Leste	0.3 (0.05, 1)	12 (2, 22)	0.6 (0.3, 1)	28 (17, 38)	16 (4, 35)	1 (0.3, 3)
Tonga	0.03 (0.004, 0.06)	15 (3, 27)	0.05 (0.03, 0.08)	35 (22, 47)	1 (0.3, 2)	0.09 (0.02, 0.2)
Tunisia	2 (0.3, 6)	14 (3, 25)	3 (1, 4)	32 (20, 44)	89 (14, 218)	9 (1, 22)
Türkiye	22 (4, 40)	17 (3, 29)	26 (14, 40)	38 (24, 50)	665 (135, 1264)	67 (13, 127)
Tuvalu	0.002 (0.0003, 0.004)	10 (2, 18)	0.003 (0.002, 0.005)	24 (15, 32)	0.07 (0.02, 0.1)	0.006 (0.002, 0.01)
Tanzania	19 (2, 49)	9 (2, 16)	44 (25, 69)	21 (13, 29)	1209 (321, 2820)	103 (24, 251)
Uganda	14 (2, 33)	8 (1, 15)	27 (15, 43)	18 (11, 26)	723 (158, 1681)	64 (13, 154)
Ukraine	2 (0.4, 5)	15 (3, 28)	5 (3, 9)	35 (22, 49)	42 (8, 85)	5 (1, 10)
Uzbekistan	7 (1, 15)	14 (2, 26)	8 (4, 13)	33 (19, 46)	367 (86, 720)	34 (7, 68)
St. Vincent and the Grenadines	0.02 (0.003, 0.04)	16 (3, 29)	0.03 (0.01, 0.04)	37 (23, 50)	1 (0.2, 2)	0.08 (0.02, 0.2)
Vietnam	18 (3, 40)	15 (3, 26)	39 (22, 58)	34 (22, 46)	738 (120, 1664)	73 (13, 167)
Vanuatu	0.09 (0.01, 0.2)	13 (2, 25)	0.1 (0.06, 0.2)	30 (15, 44)	5 (1, 12)	0.5 (0.1, 1)
Samoa	0.07 (0.01, 0.2)	16 (3, 29)	0.07 (0.04, 0.1)	37 (24, 50)	2 (0.3, 4)	0.2 (0.03, 0.4)
Kosovo	0.2 (0.04, 0.4)	16 (3, 29)	0.3 (0.1, 0.4)	38 (24, 50)	4 (1, 8)	0.5 (0.09, 1)
Yemen, Rep.	10 (1, 24)	10 (2, 21)	14 (6, 25)	24 (12, 38)	465 (98, 1120)	41 (8, 100)
South Africa	20 (3, 41)	13 (2, 24)	32 (18, 49)	31 (20, 41)	942 (213, 1908)	89 (19, 185)
Zambia	5 (1, 12)	10 (2, 17)	16 (9, 24)	22 (13, 31)	190 (47, 431)	17 (4, 40)
Zimbabwe	5 (1, 12)	11 (2, 19)	10 (5, 15)	24 (15, 34)	421 (101, 937)	37 (8, 86)

Table C: Health effects estimated for low-dose calcium supplementation intervention in 2024 for each low- and middle-income country, compared to no intervention.

DALY = disability-adjusted life year. Values in parentheses represent equal-tailed 95% uncertainty intervals.

Country	Intervention costs (USD, thousands)	Cost savings from averted healthcare (USD, thousands)	Incremental cost per adverse pregnancy outcome averted (USD)	Incremental cost per DALY averted (USD)	Net Monetary Benefits (USD, thousands)	Return on Investment	Cost savings vs. high-dose supplementation (USD, thousands)
Afghanistan	1365<br>(871, 1955)	25<br>(4, 75)	187<br>(40, 493)	169<br>(47, 1169)*	-845<br>(-1576, 397)	-0.6<br>(-0.9, 0.3)	2730<br>(1742, 3910)
Angola	2903<br>(2340, 3553)	528<br>(249, 861)	63<br>(32, 116)	86<br>(35, 359)	40680<br>(7509, 93796)	14.1<br>(2.6, 32.1)	5806<br>(4679, 7107)
Albania	74<br>(56, 95)	16<br>(6, 26)	121<br>(61, 248)	182<br>(78, 836)	1366<br>(208, 2906)	18.5<br>(2.9, 38.5)	148<br>(112, 190)
Argentina	1898<br>(1522, 2321)	1139<br>(491, 1834)	41<br>(3, 117)	37<br>(1, 236)	217231<br>(43859, 416950)	114.8<br>(23.6, 219.4)	3796<br>(3044, 4643)
Armenia	99<br>(80, 121)	25<br>(10, 40)	103<br>(52, 212)	175<br>(79, 790)	1897<br>(321, 3743)	19.2<br>(3.3, 37.7)	199<br>(159, 242)
Azerbaijan	359<br>(248, 469)	110<br>(42, 188)	88<br>(32, 202)	60<br>(18, 341)	21533<br>(3322, 50336)	60.3<br>(9.2, 139.0)	718<br>(496, 938)
Burundi	742<br>(552, 969)	11<br>(4, 20)	111<br>(57, 210)	184<br>(78, 773)*	-635<br>(-852, -445)	-0.9<br>(-1.0, -0.7)	1485<br>(1104, 1938)
Benin	865<br>(688, 1072)	67<br>(31, 114)	76<br>(43, 132)	95<br>(36, 457)	3371<br>(-34, 9620)	3.9<br>(-0.04, 11.1)	1730<br>(1376, 2143)
Burkina Faso	1287<br>(1011, 1610)	67<br>(30, 114)	83<br>(46, 146)	152<br>(66, 606)	838<br>(-758, 3467)	0.6<br>(-0.6, 2.7)	2575<br>(2021, 3220)
Bangladesh	3699<br>(2948, 4558)	686<br>(254, 1114)	69<br>(32, 148)	110<br>(49, 420)	33869<br>(6608, 72624)	9.2<br>(1.8, 19.5)	7399<br>(5896, 9117)
Bulgaria	165<br>(119, 204)	76<br>(30, 124)	75<br>(22, 193)	110<br>(29, 615)	8479<br>(1610, 16546)	51.5<br>(10.2, 99.4)	331<br>(238, 409)
Bosnia and Herzegovina	79<br>(55, 102)	20<br>(8, 32)	104<br>(51, 212)	136<br>(57, 623)	2157<br>(354, 4485)	27.4<br>(4.6, 55.7)	158<br>(110, 205)
Belarus	284<br>(234, 339)	93<br>(43, 147)	70<br>(33, 141)	202<br>(86, 896)	4884<br>(954, 9627)	17.2<br>(3.4, 33.5)	568<br>(468, 679)
Belize	23<br>(18, 27)	7<br>(3, 12)	65<br>(28, 134)	105<br>(41, 456)	652<br>(148, 1360)	29.0<br>(6.7, 60.1)	45<br>(37, 55)
Bolivia	765<br>(578, 979)	106<br>(41, 176)	120<br>(59, 243)	72<br>(34, 258)	16890<br>(4089, 35907)	22.2<br>(5.6, 45.7)	1529<br>(1157, 1958)
Brazil	8356<br>(7220, 9620)	3794<br>(1560, 6070)	52<br>(17, 127)	65<br>(20, 334)	434421<br>(98332, 816682)	52.1<br>(11.8, 98.0)	16711<br>(14439, 19240)
Bhutan	28<br>(22, 34)	5<br>(1, 13)	94<br>(18, 235)	61<br>(11, 678)	618<br>(35, 2311)	22.5<br>(1.3, 82.4)	55<br>(43, 69)
Botswana	117<br>(53, 181)	59<br>(21, 115)	33<br>(3, 83)	54<br>(3, 457)	5551<br>(657, 16639)	48.1<br>(6.4, 135.4)	233<br>(106, 361)
Central African Republic	349<br>(280, 429)	9<br>(4, 16)	100<br>(53, 182)	91<br>(39, 394)*	-25<br>(-278, 390)	-0.1<br>(-0.8, 1.1)	699<br>(560, 858)
China	33086<br>(29260, 37130)	11936<br>(5096, 18422)	99<br>(42, 220)	156<br>(58, 776)	1281427<br>(242672, 2522364)	38.9<br>(7.3, 77.3)	66172<br>(58519, 74260)
Côte d’Ivoire	1684<br>(1363, 2056)	295<br>(131, 496)	58<br>(28, 110)	71<br>(26, 349)	22152<br>(3064, 55574)	13.2<br>(1.8, 33.0)	3368<br>(2727, 4112)
Cameroon	2139<br>(1743, 2594)	225<br>(107, 369)	67<br>(37, 118)	77<br>(33, 323)	14417<br>(1742, 35090)	6.8<br>(0.8, 16.3)	4278<br>(3486, 5187)
Congo, Dem. Rep.	6271<br>(5149, 7529)	264<br>(111, 452)	83<br>(43, 153)	83<br>(35, 360)	4575<br>(-3882, 18504)	0.7<br>(-0.6, 2.9)	12542<br>(10298, 15058)
Congo, Rep.	489<br>(376, 621)	73<br>(34, 122)	66<br>(35, 120)	95<br>(42, 338)	4765<br>(999, 10785)	9.8<br>(2.2, 21.8)	979<br>(752, 1241)
Colombia	2126<br>(1676, 2637)	583<br>(238, 940)	85<br>(42, 177)	152<br>(69, 591)	42158<br>(10214, 81882)	19.9<br>(4.9, 38.0)	4252<br>(3352, 5273)
Comoros	47<br>(21, 73)	4<br>(2, 9)	71<br>(39, 125)	149<br>(64, 605)	129<br>(-3, 384)	2.7<br>(-0.1, 7.4)	94<br>(43, 146)
Cabo Verde	28<br>(22, 35)	7<br>(3, 11)	53<br>(26, 100)	113<br>(42, 560)	392<br>(57, 933)	14.0<br>(2.0, 33.0)	56<br>(45, 70)
Costa Rica	189<br>(150, 232)	102<br>(45, 163)	49<br>(9, 127)	76<br>(12, 479)	11368<br>(2253, 24176)	60.4<br>(12.2, 128.6)	378<br>(301, 464)
Cuba	261<br>(219, 308)	107<br>(55, 165)	55<br>(23, 109)	204<br>(81, 801)	5228<br>(1389, 9727)	20.1<br>(5.3, 37.4)	522<br>(439, 615)

Country	Intervention costs (USD, thousands)	Cost savings from averted healthcare (USD, thousands)	Incremental cost per adverse pregnancy outcome averted (USD)	Incremental cost per DALY averted (USD)	Net Monetary Benefits (USD, thousands)	Return on Investment	Cost savings vs. high-dose supplementation (USD, thousands)
Djibouti	63 (37, 94)	13 (6, 23)	55 (29, 101)	99 (41, 372)	788 (159, 1889)	12.5 (2.8, 28.4)	127 (75, 187)
Dominica	3 (2, 4)	1 (0.5, 2)	60 (23, 131)	82 (26, 446)	124 (21, 279)	43.4 (7.5, 95.1)	6 (4, 8)
Dominican Republic	621 (503, 754)	289 (133, 458)	50 (15, 116)	61 (17, 278)	38943 (10454, 79939)	62.9 (17.5, 127.8)	1243 (1006, 1507)
Algeria	2043 (1638, 2508)	426 (182, 705)	80 (37, 159)	53 (20, 268)	73003 (11687, 169800)	35.8 (5.6, 82.9)	4087 (3276, 5016)
Ecuador	888 (652, 1121)	302 (149, 498)	49 (21, 95)	86 (28, 379)	27398 (6272, 65158)	31.0 (7.1, 72.8)	1776 (1304, 2243)
Egypt, Arab Rep.	6295 (4102, 8201)	1235 (523, 2051)	83 (40, 163)	412 (179, 1427)	25318 (3478, 60525)	4.1 (0.6, 9.4)	12590 (8204, 16402)
Eritrea	209 (91, 349)	8 (3, 17)	88 (48, 156)	177 (86, 533)*	-7 (-155, 223)	-0.05 (-0.7, 0.9)	418 (181, 698)
Ethiopia	5766 (4729, 6935)	436 (189, 726)	71 (37, 130)	125 (53, 528)	9658 (-2163, 28596)	1.7 (-0.4, 4.9)	11531 (9458, 13870)
Fiji	52 (41, 66)	12 (5, 19)	83 (40, 163)	97 (37, 458)	1320 (235, 3044)	25.3 (4.5, 57.7)	105 (82, 131)
Micronesia, Fed. Sts.	6 (3, 10)	1 (0.4, 2)	100 (50, 189)	183 (79, 688)	57 (9, 137)	8.8 (1.7, 20.1)	13 (7, 19)
Gabon	124 (54, 203)	58 (22, 110)	38 (10, 85)	72 (15, 338)	5573 (1158, 14328)	45.5 (11.6, 106.4)	248 (109, 407)
Georgia	133 (107, 163)	28 (10, 45)	132 (67, 297)	198 (95, 781)	2192 (466, 4233)	16.5 (3.5, 31.8)	267 (213, 327)
Ghana	2608 (2135, 3142)	250 (108, 414)	105 (55, 194)	164 (67, 721)	12178 (636, 32162)	4.7 (0.2, 12.3)	5217 (4270, 6283)
Guinea	566 (426, 734)	55 (25, 93)	72 (39, 127)	86 (37, 337)	3188 (382, 7791)	5.6 (0.7, 13.6)	1133 (852, 1467)
Gambia, The	238 (186, 299)	13 (6, 21)	83 (46, 146)	141 (63, 505)	189 (-119, 702)	0.8 (-0.5, 2.9)	477 (373, 597)
Guinea-Bissau	177 (141, 219)	9 (4, 15)	84 (46, 148)	123 (54, 485)	146 (-96, 539)	0.8 (-0.5, 3.0)	355 (282, 438)
Equatorial Guinea	99 (42, 169)	42 (15, 81)	38 (13, 82)	63 (17, 313)	4059 (735, 10337)	41.7 (9.3, 94.0)	197 (84, 337)
Grenada	6 (4, 7)	3 (1, 5)	37 (9, 86)	64 (14, 277)	300 (89, 607)	53.2 (16.4, 105.2)	11 (8, 15)
Guatemala	1089 (893, 1310)	232 (72, 420)	126 (38, 314)	130 (50, 447)	21442 (6183, 47231)	19.7 (5.7, 43.3)	2179 (1785, 2621)
Honduras	646 (485, 832)	79 (32, 130)	115 (59, 223)	146 (71, 472)	5584 (1256, 11964)	8.7 (2.1, 18.0)	1291 (970, 1663)
Haiti	598 (453, 768)	46 (19, 75)	116 (62, 216)	155 (85, 362)	2121 (552, 4485)	3.6 (1.0, 7.3)	1196 (905, 1535)
Indonesia	11504 (9920, 13249)	2453 (1174, 3894)	78 (39, 148)	86 (36, 322)	289919 (71547, 624012)	25.3 (6.4, 54.6)	23007 (19840, 26499)
India	45306 (39351, 51790)	6937 (2911, 10797)	73 (38, 142)	64 (28, 279)	649173 (103230, 1385636)	14.4 (2.3, 30.5)	90613 (78702, 103580)
Iran, Islamic Rep.	2872 (1858, 3807)	510 (216, 853)	96 (47, 186)	138 (53, 676)	41312 (5346, 99039)	14.5 (1.8, 33.7)	5744 (3715, 7615)
Iraq	2802 (2244, 3444)	720 (300, 1182)	83 (35, 174)	106 (38, 552)	71605 (11449, 165666)	25.6 (4.0, 59.2)	5604 (4488, 6887)
Jordan	741 (578, 928)	114 (41, 195)	53 (55, 280)	156 (61, 764)	9057 (1105, 21718)	12.2 (1.5, 29.0)	1483 (1157, 1856)
Kazakhstan	1246 (1051, 1458)	473 (235, 732)	72 (32, 145)	141 (55, 646)	44510 (9346, 87343)	35.8 (7.6, 71.0)	2492 (2102, 2915)
Kenya	2993 (2419, 3654)	380 (185, 613)	68 (38, 119)	143 (67, 486)	15034 (2367, 33981)	5.0 (0.8, 11.3)	5986 (4838, 7308)
Kyrgyz Republic	478 (399, 565)	26 (11, 41)	164 (92, 310)	124 (62, 475)	1990 (107, 4331)	4.2 (0.2, 9.0)	957 (798, 1130)

Country	Intervention costs (USD, thousands)	Cost savings from averted healthcare (USD, thousands)	Incremental cost per adverse pregnancy outcome averted (USD)	Incremental cost per DALY averted (USD)	Net Monetary Benefits (USD, thousands)	Return on Investment	Cost savings vs. high-dose supplementation (USD, thousands)
Cambodia	838<br>(441, 1198)	65<br>(25, 120)	104<br>(55, 187)	154<br>(60, 683)	3112<br>(26, 9142)	3.7<br>(0.04, 10.4)	1676<br>(883, 2397)
Kiribati	8<br>(6, 10)	0.6<br>(0.2, 0.9)	122<br>(64, 225)	100<br>(40, 491)	46<br>(2, 117)	5.8<br>(0.2, 14.9)	16<br>(13, 19)
Lao PDR	334<br>(258, 422)	36<br>(14, 69)	90<br>(39, 174)	66<br>(19, 528)	4041<br>(157, 12403)	12.1<br>(0.5, 36.5)	668<br>(515, 845)
Lebanon	181<br>(110, 260)	31<br>(11, 57)	105<br>(44, 220)	167<br>(57, 947)	1979<br>(163, 5365)	11.0<br>(0.9, 28.9)	362<br>(220, 521)
Liberia	497<br>(396, 613)	23<br>(11, 40)	84<br>(46, 148)	152<br>(71, 484)	209<br>(-274, 1001)	0.4<br>(-0.5, 2.0)	994<br>(792, 1225)
Libya	296<br>(183, 416)	102<br>(41, 189)	54<br>(17, 117)	118<br>(27, 726)	7009<br>(1024, 19763)	23.8<br>(3.6, 66.0)	592<br>(366, 833)
St. Lucia	6<br>(4, 9)	3<br>(1, 5)	41<br>(8, 102)	52<br>(8, 311)	447<br>(86, 998)	75.2<br>(15.4, 160.4)	12<br>(8, 17)
Sri Lanka	493<br>(74, 966)	78<br>(11, 177)	82<br>(40, 155)	214<br>(80, 947)	3072<br>(151, 9932)	6.3<br>(0.7, 16.3)	986<br>(149, 1932)
Lesotho	153<br>(120, 191)	11<br>(5, 18)	80<br>(43, 141)	82<br>(36, 325)	536<br>(17, 1389)	3.5<br>(0.1, 9.1)	305<br>(239, 381)
Morocco	1136<br>(856, 1464)	181<br>(76, 305)	93<br>(45, 183)	114<br>(47, 482)	15145<br>(2657, 35026)	13.4<br>(2.4, 30.5)	2271<br>(1711, 2928)
Moldova	138<br>(100, 167)	27<br>(13, 43)	95<br>(52, 171)	282<br>(133, 1138)	1244<br>(192, 2614)	9.1<br>(1.4, 18.8)	275<br>(199, 335)
Madagascar	1580<br>(1216, 2010)	49<br>(22, 86)	93<br>(50, 166)	170<br>(78, 587)*	-508<br>(-1328, 693)	-0.3<br>(-0.8, 0.4)	3159<br>(2432, 4019)
Maldives	18<br>(14, 23)	11<br>(5, 17)	35<br>(4, 94)	66<br>(5, 404)	954<br>(208, 1943)	53.2<br>(11.9, 106.8)	36<br>(28, 45)
Mexico	5871<br>(4890, 6953)	3318<br>(1739, 5123)	32<br>(7, 75)	73<br>(13, 357)	283031<br>(71469, 541889)	48.4<br>(12.4, 93.1)	11742<br>(9780, 13906)
Marshall Islands	2<br>(1, 3)	0.5<br>(0.2, 0.9)	85<br>(38, 174)	95<br>(36, 406)	68<br>(14, 159)	33.8<br>(7.6, 74.4)	4<br>(2, 6)
North Macedonia	63<br>(51, 76)	13<br>(6, 21)	114<br>(62, 219)	106<br>(49, 470)	1960<br>(332, 3842)	31.2<br>(5.4, 61.1)	126<br>(102, 152)
Mali	1420<br>(1097, 1799)	61<br>(28, 107)	97<br>(53, 169)	167<br>(65, 791)	722<br>(-976, 3877)	0.5<br>(-0.7, 2.7)	2840<br>(2194, 3599)
Myanmar	1780<br>(1376, 2250)	94<br>(42, 160)	105<br>(58, 185)	109<br>(48, 436)	4270<br>(-291, 11778)	2.4<br>(-0.2, 6.6)	3559<br>(2752, 4500)
Mongolia	195<br>(164, 229)	28<br>(11, 49)	146<br>(62, 299)	179<br>(63, 961)	2594<br>(289, 6754)	13.3<br>(1.5, 34.5)	390<br>(328, 459)
Mozambique	2105<br>(1660, 2626)	62<br>(29, 106)	100<br>(57, 174)	160<br>(73, 591)*	-410<br>(-1690, 1545)	-0.2<br>(-0.8, 0.7)	4210<br>(3320, 5252)
Mauritania	341<br>(246, 455)	49<br>(22, 83)	62<br>(34, 111)	90<br>(39, 350)	3016<br>(505, 7178)	8.9<br>(1.6, 20.5)	683<br>(491, 910)
Mauritius	26<br>(12, 40)	14<br>(5, 25)	36<br>(5, 90)	52<br>(6, 330)	1669<br>(274, 4136)	65.6<br>(12.1, 148.3)	52<br>(23, 80)
Malawi	1170<br>(871, 1524)	62<br>(27, 108)	69<br>(37, 125)	122<br>(55, 473)	442<br>(-756, 2282)	0.4<br>(-0.6, 1.9)	2340<br>(1742, 3047)
Malaysia	1656<br>(1319, 2032)	946<br>(468, 1539)	35<br>(4, 86)	139<br>(15, 735)	44843<br>(10817, 97289)	27.2<br>(6.7, 58.3)	3312<br>(2638, 4063)
Namibia	135<br>(60, 213)	39<br>(14, 71)	53<br>(24, 104)	115<br>(42, 510)	2353<br>(398, 5904)	17.7<br>(3.5, 40.2)	269<br>(120, 426)
Niger	1577<br>(1233, 1980)	49<br>(21, 87)	98<br>(53, 177)	210<br>(93, 826)*	-620<br>(-1385, 514)	-0.4<br>(-0.8, 0.3)	3155<br>(2466, 3960)
Nigeria	15749<br>(13485, 18238)	2342<br>(1105, 3860)	61<br>(31, 111)	63<br>(23, 321)	204259<br>(23980, 510396)	13.0<br>(1.5, 32.2)	31497<br>(26970, 36476)
Nicaragua	411<br>(285, 553)	31<br>(14, 51)	136<br>(85, 231)	308<br>(170, 981)	930<br>(3, 2090)	2.3<br>(0.006, 4.9)	822<br>(569, 1106)
Nepal	1594<br>(1249, 1992)	108<br>(42, 181)	111<br>(55, 220)	238<br>(121, 675)	1722<br>(-417, 4898)	1.1<br>(-0.3, 3.0)	3188<br>(2498, 3983)

Country	Intervention costs (USD, thousands)	Cost savings from averted healthcare (USD, thousands)	Incremental cost per adverse pregnancy outcome averted (USD)	Incremental cost per DALY averted (USD)	Net Monetary Benefits (USD, thousands)	Return on Investment	Cost savings vs. high-dose supplementation (USD, thousands)
Pakistan	11490 (9731, 13434)	1241 (477, 2093)	80 (37, 163)	56 (25, 196)	111206 (23469, 249616)	9.7 (2.1, 21.7)	22980 (19462, 26867)
Peru	1884 (1457, 2363)	352 (119, 593)	168 (81, 377)	201 (101, 659)	34310 (9522, 66510)	18.3 (5.4, 34.6)	3767 (2915, 4727)
Philippines	7316 (6196, 8566)	1278 (601, 2107)	75 (37, 140)	128 (50, 528)	83618 (15191, 203337)	11.5 (2.1, 27.7)	14633 (12392, 17131)
Palau	0.7 (0.4, 1)	0.3 (0.1, 1)	50 (12, 119)	100 (18, 601)	32 (5, 75)	45.7 (8.3, 103.1)	1 (1, 2)
Papua New Guinea	420 (295, 570)	58 (22, 101)	101 (47, 201)	84 (34, 368)	6419 (1078, 15467)	15.3 (2.7, 36.4)	840 (590, 1140)
Paraguay	395 (295, 512)	98 (43, 159)	84 (45, 164)	137 (66, 499)	8171 (2041, 15938)	20.8 (5.4, 39.4)	790 (590, 1023)
Russian Federation	4104 (2935, 5106)	2429 (1200, 3862)	40 (4, 99)	188 (16, 939)	105990 (25997, 200417)	26.0 (6.6, 48.5)	8208 (5869, 10213)
Rwanda	656 (512, 824)	40 (16, 75)	80 (38, 150)	102 (37, 441)	1325 (-187, 4579)	2.0 (-0.3, 6.8)	1312 (1024, 1648)
Sudan	3080 (1406, 4739)	149 (44, 295)	143 (69, 284)	46 (19, 212)	22067 (1548, 61029)	7.1 (0.5, 17.5)	6160 (2812, 9477)
Senegal	1062 (829, 1331)	107 (50, 178)	71 (39, 125)	122 (53, 480)	4248 (296, 10757)	4.0 (0.3, 10.0)	2124 (1657, 2662)
Solomon Islands	50 (39, 63)	4 (2, 7)	121 (62, 227)	180 (85, 549)	216 (38, 507)	4.3 (0.8, 10.0)	100 (78, 125)
Sierra Leone	709 (546, 898)	20 (9, 35)	95 (51, 170)	106 (47, 380)*	-55 (-542, 703)	-0.1 (-0.7, 1.0)	1418 (1092, 1795)
El Salvador	297 (212, 387)	62 (27, 99)	92 (50, 176)	170 (80, 660)	4074 (817, 8352)	13.8 (2.8, 27.6)	594 (424, 775)
Somalia	1536 (701, 2357)	45 (14, 91)	92 (50, 165)	173 (75, 713)*	-653 (-1543, 406)	-0.4 (-0.9, 0.3)	3072 (1403, 4714)
South Sudan	636 (273, 1072)	44 (14, 89)	76 (42, 135)	118 (49, 520)	1285 (-207, 4264)	2.0 (-0.4, 5.9)	1272 (546, 2144)
São Tomé and Prín cipe	13 (6, 20)	2 (1, 4)	59 (32, 105)	185 (79, 716)	56 (5, 152)	4.5 (0.5, 10.9)	25 (12, 40)
Suriname	25 (20, 31)	9 (4, 15)	54 (18, 125)	45 (13, 239)	1322 (256, 2997)	52.7 (10.1, 119.0)	50 (40, 62)
Eswatini	55 (25, 87)	14 (5, 25)	55 (26, 103)	72 (26, 357)	1269 (181, 3270)	23.3 (3.8, 54.1)	110 (49, 174)
Syrian Arab Republic	1369 (836, 1950)	35 (13, 67)	128 (66, 242)	278 (116, 1276)*	-727 (-1425, 74)	-0.5 (-0.9, 0.1)	2738 (1671, 3899)
Chad	829 (601, 1106)	38 (16, 66)	86 (47, 152)	92 (38, 404)	953 (-438, 3305)	1.1 (-0.5, 3.9)	1658 (1203, 2212)
Togo	525 (414, 654)	30 (14, 51)	81 (45, 143)	124 (51, 544)	695 (-263, 2281)	1.3 (-0.5, 4.3)	1050 (829, 1309)
Thailand	1863 (1417, 2375)	639 (311, 1043)	53 (23, 105)	176 (65, 743)	30313 (6483, 66709)	16.3 (3.5, 35.4)	3725 (2834, 4750)
Tajikistan	552 (431, 691)	19 (8, 31)	184 (102, 349)	273 (132, 1106)	180 (-405, 926)	0.3 (-0.7, 1.7)	1104 (863, 1383)
Turkmenistan	422 (337, 517)	99 (39, 160)	120 (58, 253)	66 (28, 295)	23396 (4520, 47562)	55.6 (11.1, 112.8)	844 (674, 1034)
Timor-Leste	85 (61, 114)	9 (4, 16)	92 (50, 166)	142 (64, 510)	527 (85, 1255)	6.2 (1.1, 14.3)	171 (122, 229)
Tonga	7 (6, 9)	1 (1, 2)	84 (42, 159)	157 (66, 593)	92 (19, 208)	12.5 (2.7, 28.1)	15 (11, 19)
Tunisia	520 (424, 629)	85 (34, 146)	99 (43, 204)	117 (40, 648)	7402 (778, 19068)	14.3 (1.5, 36.2)	1041 (849, 1258)
Türkiye	3612 (2929, 4380)	2033 (876, 3220)	38 (6, 104)	54 (7, 338)	221856 (45045, 431624)	61.6 (12.6, 119.9)	7225 (5859, 8760)
Tuvalu	0.5 (0.4, 0.6)	0.1 (0.05, 0.2)	86 (41, 168)	154 (63, 627)	8 (2, 19)	15.8 (3.4, 35.4)	1 (0.8, 1)

Country	Intervention costs (USD, thousands)	Cost savings from averted healthcare (USD, thousands)	Incremental cost per adverse pregnancy outcome averted (USD)	Incremental cost per DALY averted (USD)	Net Monetary Benefits (USD, thousands)	Return on Investment	Cost savings vs. high-dose supplementation (USD, thousands)
Tanzania	5061 (4212, 6018)	356 (162, 608)	81 (43, 145)	119 (50, 449)	12503 (-337, 36022)	2.5 (-0.1, 7.1)	10123 (8424, 12035)
Uganda	3317 (2699, 4028)	200 (90, 335)	83 (45, 147)	131 (56, 552)	4465 (-1487, 14407)	1.3 (-0.5, 4.3)	6635 (5398, 8056)
Ukraine	651 (457, 831)	138 (66, 223)	72 (39, 132)	183 (85, 757)	6918 (1090, 14469)	10.7 (1.7, 21.8)	1303 (915, 1662)
Uzbekistan	2226 (1590, 2772)	172 (70, 276)	147 (83, 274)	155 (78, 581)	11996 (1263, 26044)	5.4 (0.6, 11.5)	4451 (3179, 5544)
St. Vincent and the Grenadines	4 (3, 5)	2 (1, 3)	58 (21, 129)	72 (23, 331)	201 (49, 422)	52.0 (12.9, 107.1)	8 (6, 10)
Vietnam	4150 (3483, 4889)	968 (472, 1539)	60 (30, 113)	106 (41, 533)	68095 (9741, 157498)	16.5 (2.3, 37.9)	8299 (6966, 9779)
Vanuatu	26 (14, 37)	3 (1, 6)	109 (55, 206)	124 (53, 500)	264 (40, 644)	10.2 (1.7, 23.6)	52 (27, 74)
Samoa	20 (16, 24)	2 (1, 4)	137 (65, 276)	235 (95, 1082)	134 (12, 330)	6.9 (0.6, 16.8)	39 (32, 48)
Kosovo	60 (47, 74)	11 (5, 18)	110 (60, 210)	219 (105, 919)	678 (102, 1364)	11.4 (1.7, 22.7)	119 (94, 148)
Yemen, Rep.	2614 (1662, 3545)	89 (34, 161)	122 (63, 230)	167 (73, 688)	441 (-1921, 4274)	0.2 (-0.7, 1.6)	5227 (3324, 7090)
South Africa	2867 (2296, 3519)	1416 (661, 2238)	31 (9, 74)	41 (9, 222)	156784 (33351, 328213)	54.9 (11.8, 114.6)	5735 (4592, 7038)
Zambia	1506 (1159, 1906)	138 (66, 230)	70 (40, 120)	202 (89, 746)	2695 (-328, 7768)	1.8 (-0.2, 5.1)	3011 (2319, 3812)
Zimbabwe	1196 (971, 1454)	91 (41, 151)	82 (44, 148)	79 (34, 326)	5551 (396, 13919)	4.6 (0.3, 11.5)	2391 (1941, 2907)

Table D: Intervention costs, cost savings, and cost-effectiveness of the low-dose calcium supplementation intervention in 2024 for each low- and middle-income country, compared to no intervention.

USD = 2022 US dollars, DALY = disability-adjusted life year. Values in parentheses represent equal-tailed 95% uncertainty intervals. Negative values for net monetary benefit or return on investment indicates that the monetary value of health gains is less than incremental costs (i.e., intervention not cost-effective at willingness to pay threshold used for analysis). *Intervention not cost-effective at country-specific cost-effectiveness threshold.

Parameter	Change in incremental cost per DALY averted (%)		Change in net monetary benefits (%)	
	Low parameter value	High parameter value	Low parameter value	High parameter value
ANC coverage	-1	0.1	-10	7
Number of livebirths for 2024	-1	1	-11	11
Proportion of births that are preterm	53	-32	-24	29
Proportion of births with preeclampsia	7	-8	-3	4
Risk ratio of preterm birth with vs. without calcium supplementation	-44	421	64	-84
Risk ratio of preeclampsia with vs. without calcium supplementation	-5	8	2	-3
Intervention adherence	34	-19	-21	16
Infant deaths per preterm birth	21	-14	-14	14
Maternal deaths per preeclampsia case	2	-2	-1	1
Infant YLLs (Years of Life Lost) per preterm birth	6	-3	-5	3
Maternal YLLs per preeclampsia case	1	-1	-1	1
Infant YLDs (Years Lived with Disability) per preterm birth	1	-1	-2	2
Maternal YLDs per preterm birth	2	-3	-4	5
Maternal YLDs per preeclampsia case	1	-1	-1	2
Unit cost of additional health services per preterm birth without death, as compared to a full term delivery	5	-8	-0.2	0.3
Unit cost of additional health services per preterm birth with death, as compared to a full term delivery	0.3	-0.5	-0.01	0.02
Unit cost of additional health services per pre-eclampsia episode, as compared to no eclampsia	4	-7	-0.2	0.3
Mark-up for supply chain	-9	8	0.4	-0.3
Wastage rate	-4	6	0.2	-0.2
Willingness to pay threshold	0	0	-22	22

Table E: Results of one-way sensitivity analyses for each parameter, for the incremental cost-effectiveness ratio (ICER) and net monetary benefit (NMB) across all low- and middle-income countries.

DALY = disability-adjusted life year. ANC = antenatal care. YLLs = years of life lost. YLDs = years lived with disability.

	Preterm births averted (thousands)	Preterm births averted (% of base-case)	Preeclampsia cases averted (thousands)	Preeclampsia cases averted (% of base-case)	Infant and maternal deaths averted (thousands)	DALYs averted (thousands)
Main analysis	1259 (209, 2681)	10 (2, 18)	1835 (1036, 2836)	23 (14, 32)	65 (15, 137)	5930 (1301, 12877)
High adherence scenario	1841 (318, 3639) *46%*	15 (3, 26) *49%*	2766 (1567, 4274) *51%*	34 (22, 45) *50%*	97 (24, 190) *49%*	8826 (2049, 17796) *49%*
High adherence and full coverage scenario	2958 (507, 5867) *135%*	24 (4, 41) *138%*	4423 (2537, 6731) *141%*	55 (36, 70) *140%*	162 (41, 316) *150%*	14650 (3418, 29626) *147%*
50% efficacy for recipients with low adherence	1550 (264, 3158) *23%*	13 (2, 22) *24%*	2300 (1311, 3517) *25%*	29 (18, 38) *25%*	81 (20, 163) *25%*	7378 (1700, 15274) *24%*
Alternative risk ratios for efficacy of calcium supplementation	894 (-4851, 4921) *-29%*	7 (-38, 34) *-29%*	2069 (1299, 3047) *13%*	26 (19, 33) *13%*	49 (-215, 238) *-24%*	4429 (-20174, 22726) *-25%*
Exclude cost-savings of averted care for pregnancy complications	1259 (209, 2681) 0%	10 (2, 18) 0%	1835 (1036, 2836) 0%	23 (14, 32) 0%	65 (15, 137) 0%	5930 (1301, 12877) 0%
Calcium unit cost $0.015	1259 (209, 2681) 0%	10 (2, 18) 0%	1835 (1036, 2836) 0%	23 (14, 32) 0%	65 (15, 137) 0%	5930 (1301, 12877) 0%
Calcium unit cost $0.03	1259 (209, 2681) 0%	10 (2, 18) 0%	1835 (1036, 2836) 0%	23 (14, 32) 0%	65 (15, 137) 0%	5930 (1301, 12877) 0%

Table F: Health impact results with alternative analytic assumptions.

DALYs = disability-adjusted life years. Values in red italics report percent difference compared to main analysis.

	Intervention costs (USD, mil.)	Cost savings from averted healthcare (USD, mil.)	Cost per adverse pregnancy outcome averted (USD)	Incremental cost per DALY averted (USD)*	Net monetary benefits (USD, mil.)	Return on investment	Cost savings vs. high-dose supplementation (USD, mil.)
Main analysis	267 (220, 318)	56 (26, 86)	75 (38, 144)	90 (38, 389)	5063 (1017, 10417)	19.1 (3.8, 39.5)	534 (440, 637)
High adherence scenario*	---<br>*---*	---<br>*---*	---<br>*---*	---<br>*---*	---<br>*---*	---<br>*---*	---<br>*---*
High adherence and full coverage scenario*	---<br>---	---<br>---	---<br>---	---<br>---	---<br>---	---<br>---	---<br>---
50% efficacy for recipients with low adherence	267 (220, 318)<br>*0%*	67 (31, 103)<br>*20%*	56 (29, 107)<br>*-25%*	69 (30, 287)<br>*-24%*	6024 (1258, 11987)<br>*19%*	22.7 (4.7, 45.2)<br>*19%*	534 (440, 637)<br>*0%*
Alternative risk ratios for efficacy of calcium supplementation	267 (220, 318)<br>*0%*	50 (-95, 138)<br>*-11%*	12 (Dom., 756)<br>*-83%*	123 (Dom., 788)<br>*36%*	3717 (-18288, 18869)<br>*-27%*	14.0 (-68.2, 72.0)<br>*-27%*	534 (440, 637)<br>*0%*
Exclude cost-savings of averted care for pregnancy complications	267 (220, 318)<br>*0%*	0 (0, 0)<br>*-100%*	93 (54, 161)<br>*25%*	114 (54, 438)<br>*26%*	5008 (979, 10340)<br>*-1%*	18.9 (3.7, 39.2)<br>*-1%*	534 (440, 637)<br>*0%*
Calcium unit cost $0.015	200 (165, 239)<br>*-25%*	56 (26, 86)<br>*0%*	51 (24, 103)<br>*-31%*	62 (24, 280)<br>*-31%*	5130 (1080, 10487)<br>*1%*	25.8 (5.4, 52.9)<br>*35%*	400 (330, 478)<br>*-25%*
Calcium unit cost $0.03	400 (330, 478)<br>*50%*	56 (26, 86)<br>*0%*	121 (65, 225)<br>*62%*	147 (65, 609)<br>*63%*	4930 (880, 10277)<br>*-3%*	12.4 (2.2, 26.0)<br>*-35%*	801 (660, 955)<br>*50%*

Table G: Cost and cost-effectiveness results with alternative analytic assumptions.

mil. = millions. DALYs = disability-adjusted life years. USD = 2022 US dollars. Values in red italics report percent difference compared to main analysis. Cost and cost-effectiveness results not calculated for high adherence and full coverage scenarios as these analyses did not consider the costs of achieving higher coverage and/or adherence.

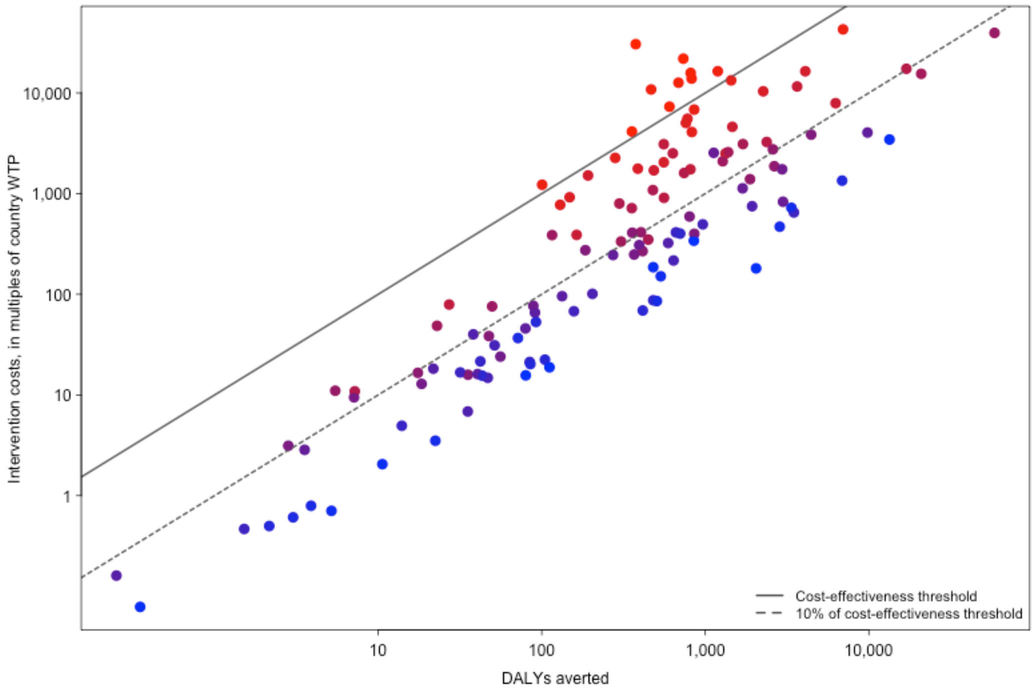

Fig B: Country-specific health benefits and intervention costs (excluding cost-savings) compared to cost-effectiveness thresholds for each country.

DALYs = disability-adjusted life years. WTP = willingness to pay. Color of points indicates country income level: warmer (red) colors indicate lower per capita GDP, cooler (blue) colors indicate higher per capita GDP.

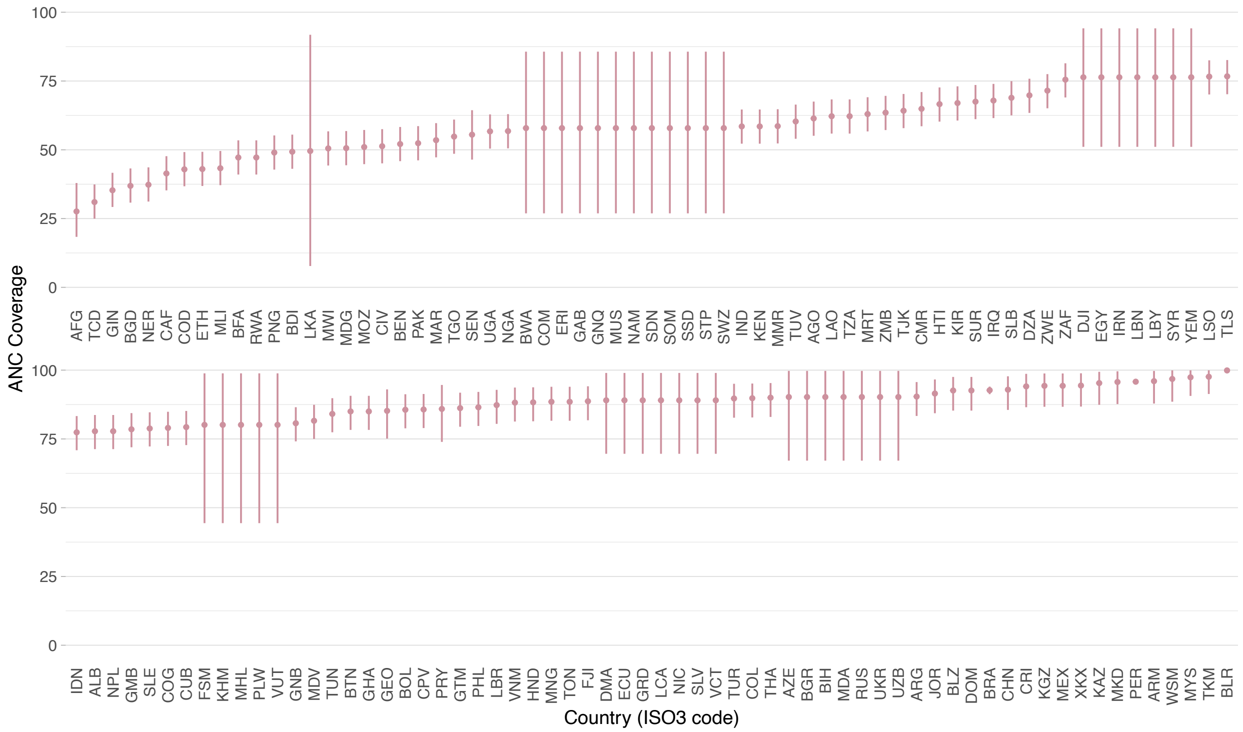

Fig C-1: Country-specific input values (ANC coverage (b_i)).

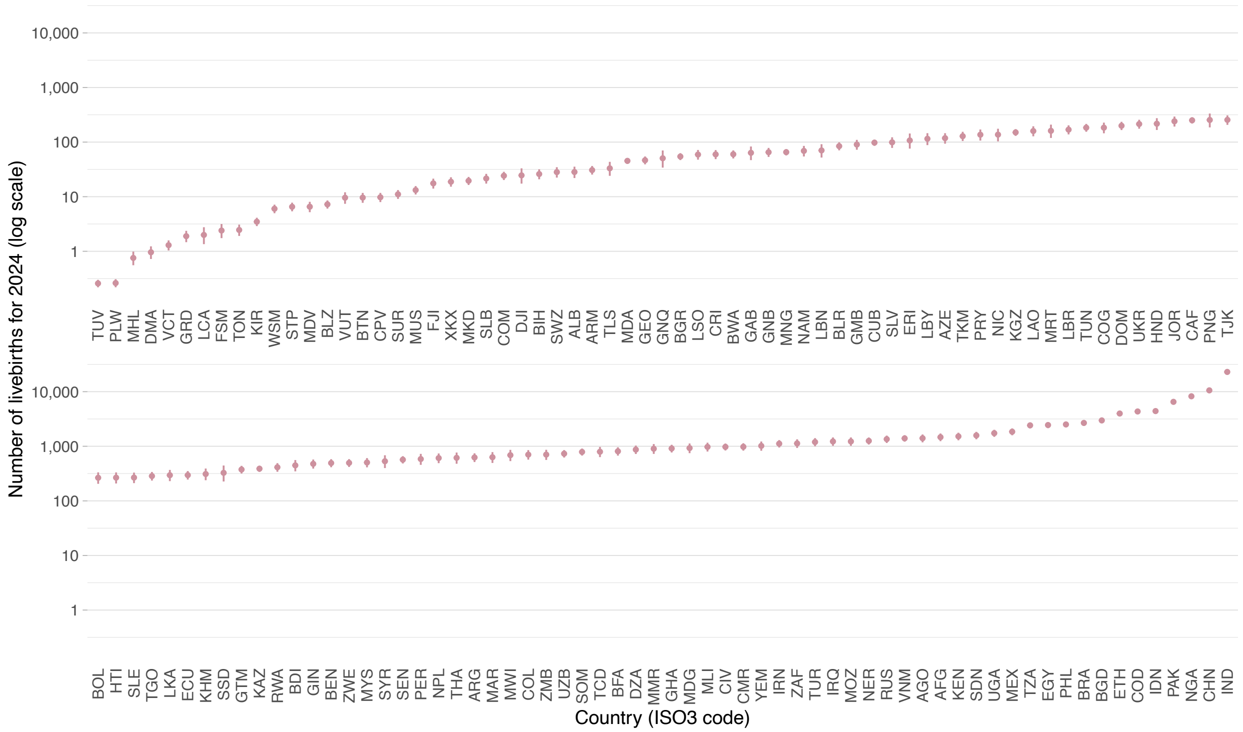

Fig C-2: Country-specific input values (number of livebirths (c_i)).

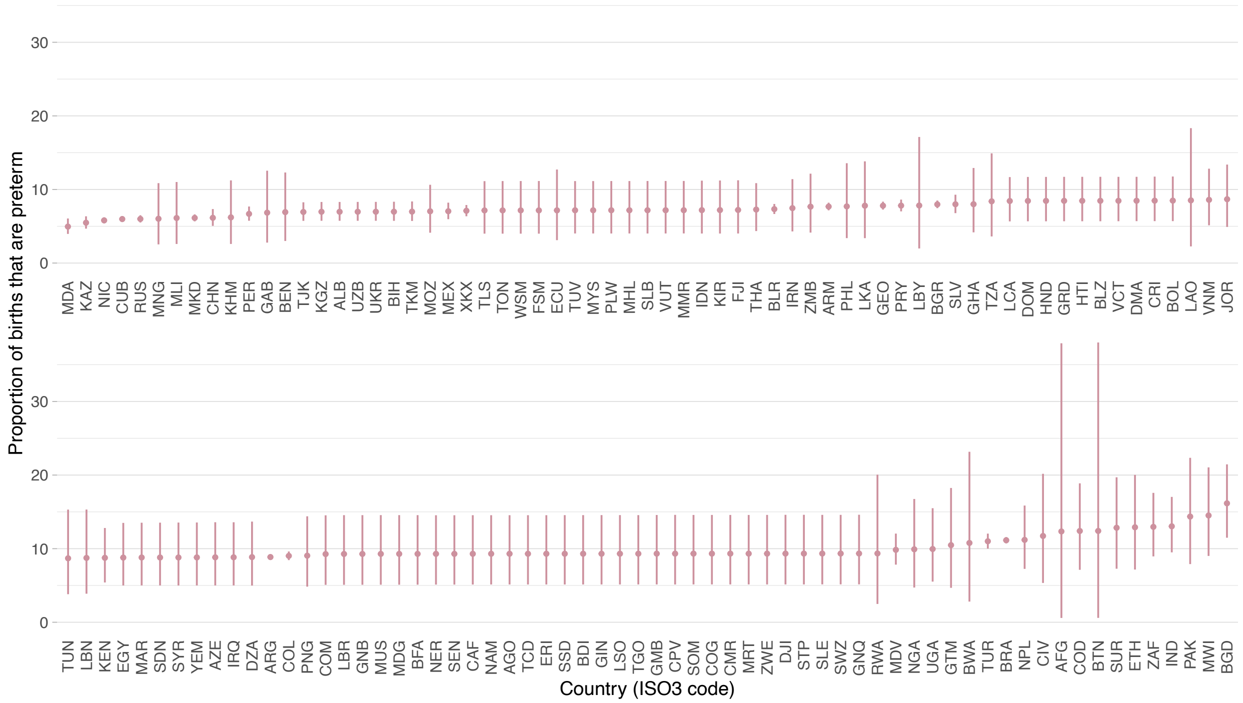

Fig C-3: Country-specific input values (proportion of births that are preterm (d_i)).

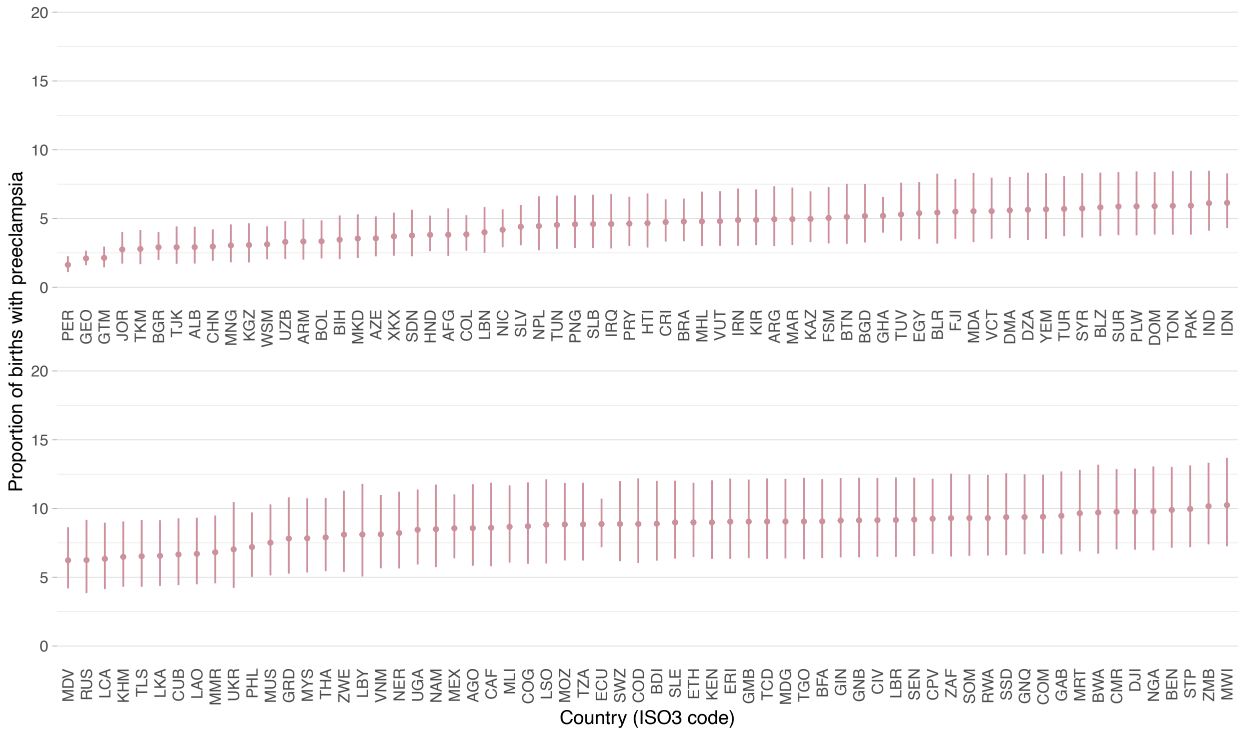

Fig C-4: Country-specific input values (proportion of births with preeclampsia (e_i)).

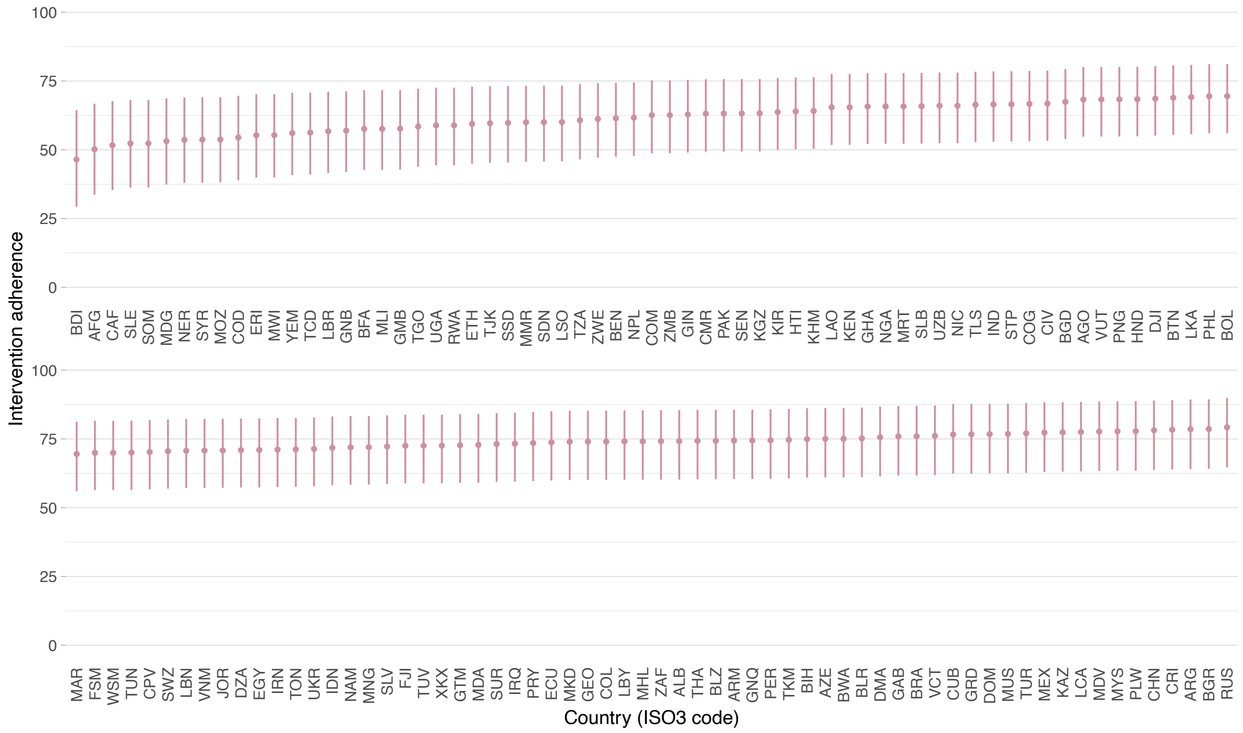

Fig C-5: Country-specific input values (intervention adherence (h_i)).

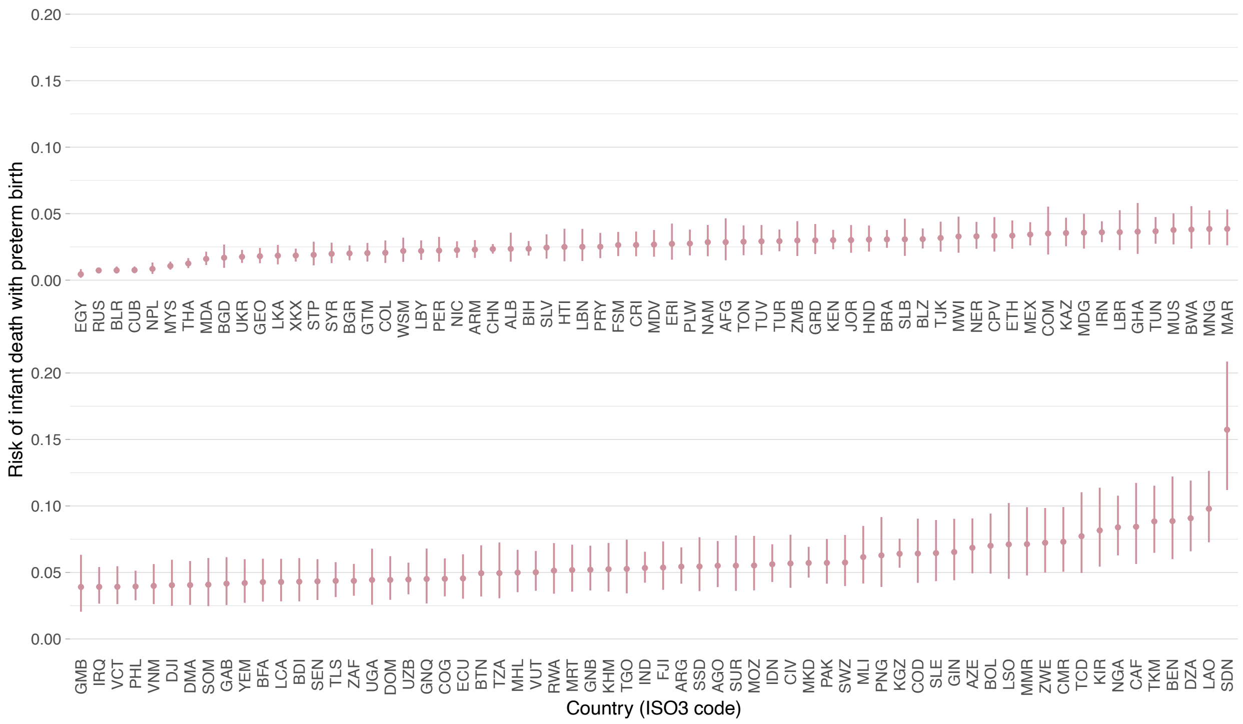

Fig C-6: Country-specific input values (infant deaths per preterm birth ($m_i^{inf\_ptb}$)).

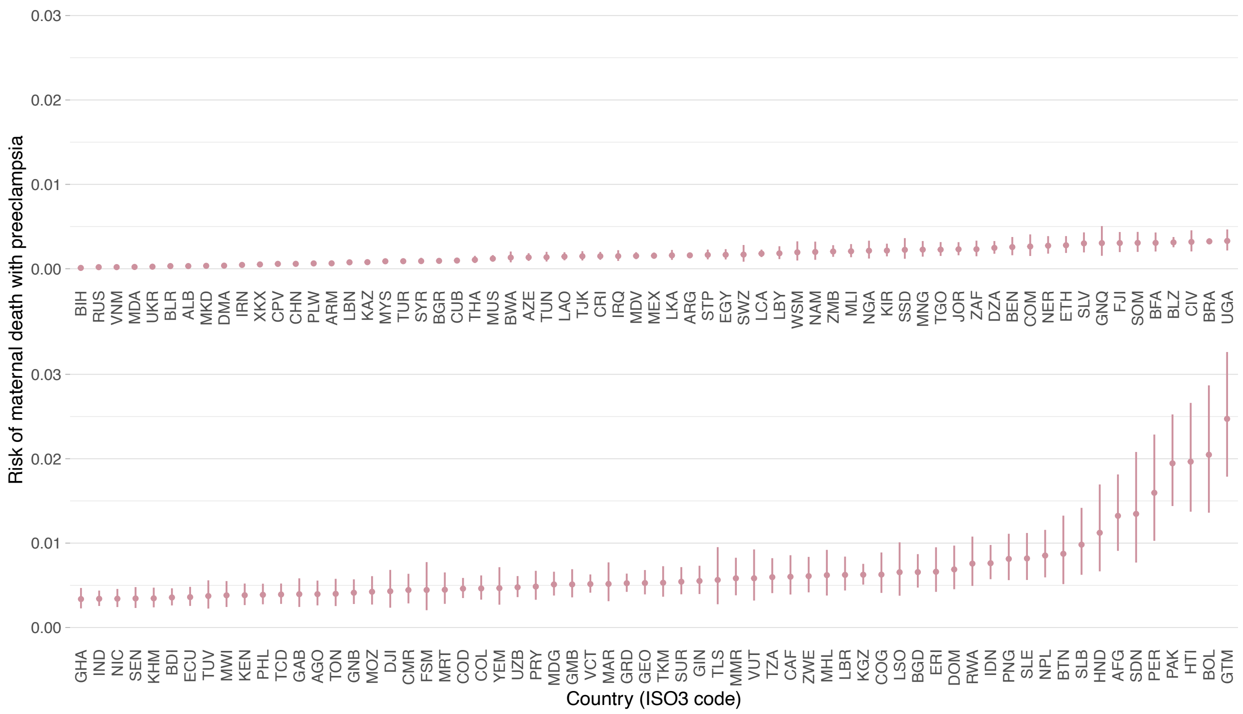

Fig C-7: Country-specific input values (maternal deaths per preeclampsia case ($m_i^{mat\_pe}$)).

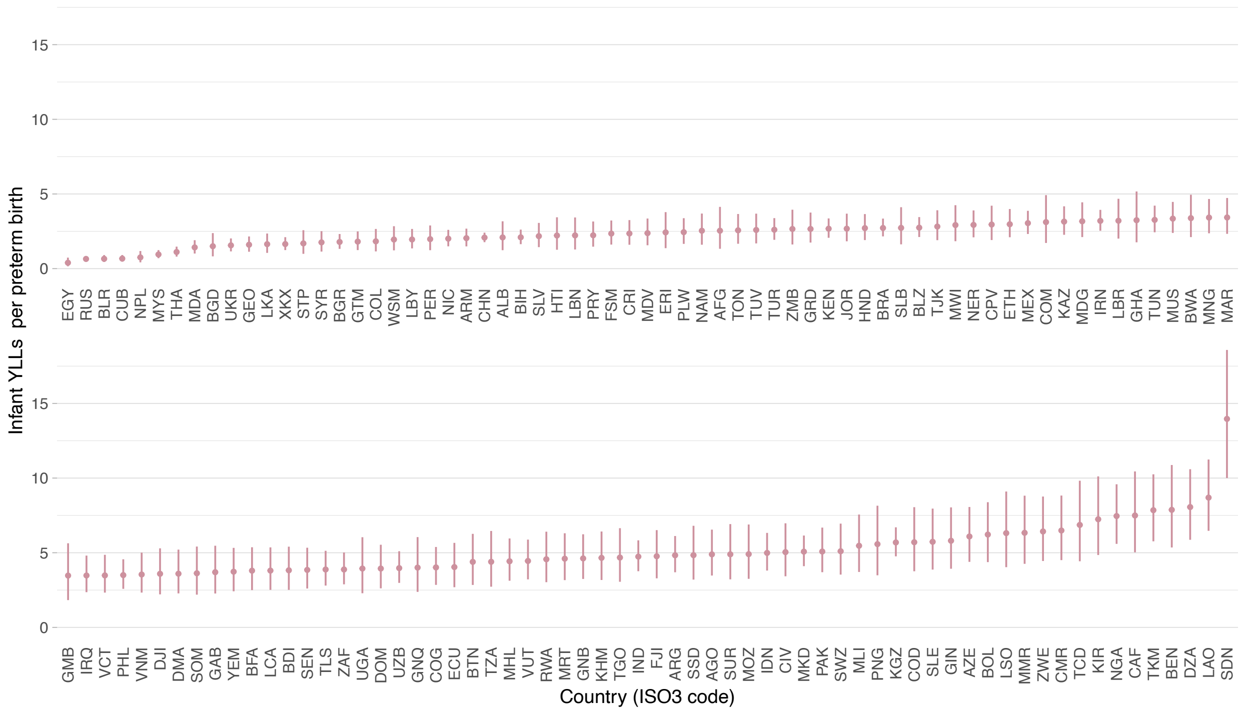

Fig C-8: Country-specific input values (infant YLLs per preterm birth ($n_i^{inf\_ptb}$)).

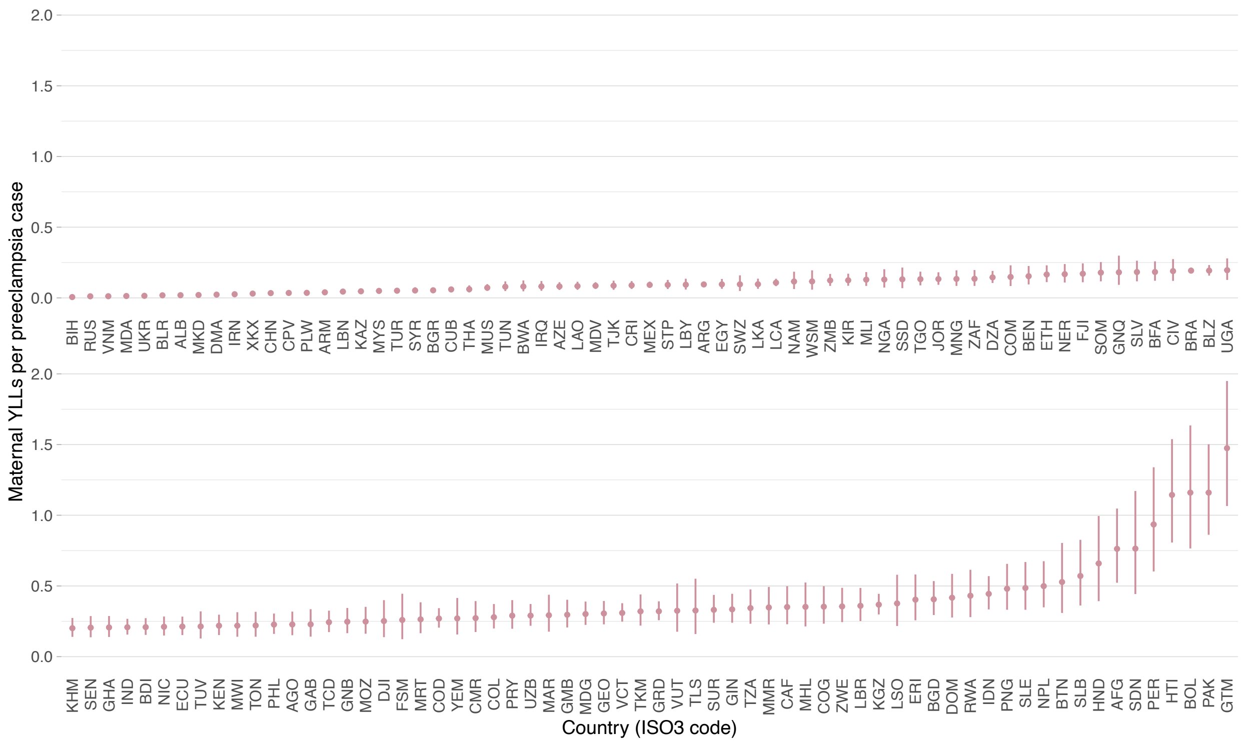

Fig C-9: Country-specific input values (maternal YLLs per preeclampsia case ($n_i^{mat\_pe}$)).

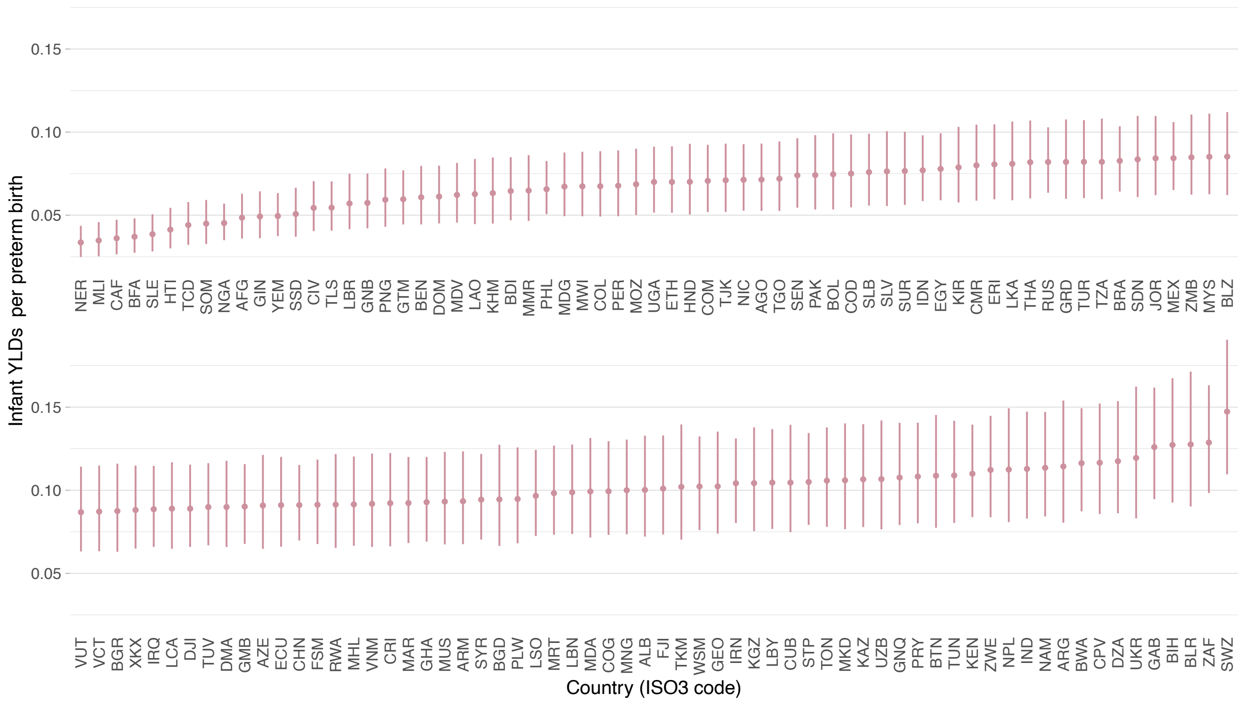

Fig C-10: Country-specific input values (infant YLDs per preterm birth ($o_i^{inf\_ptb}$)).

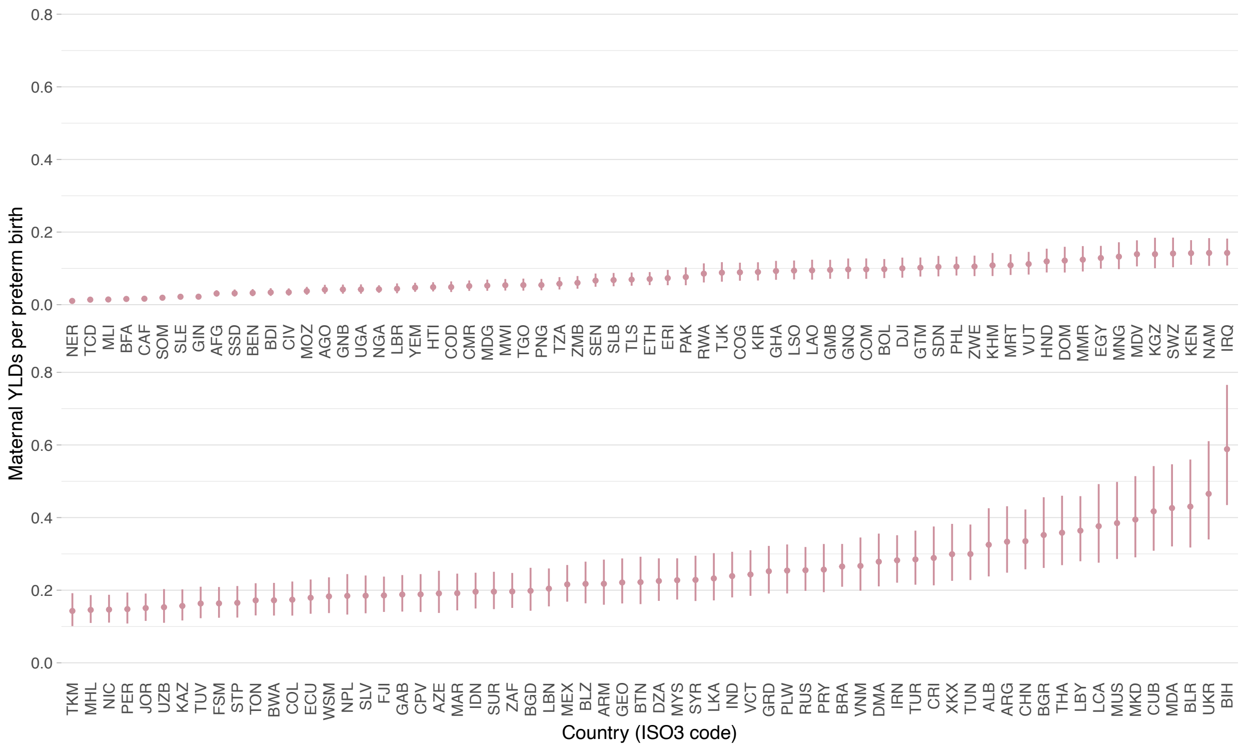

Fig C-11: Country-specific input values (maternal YLDs per preterm birth ($o_i^{mat\_ptb}$)).

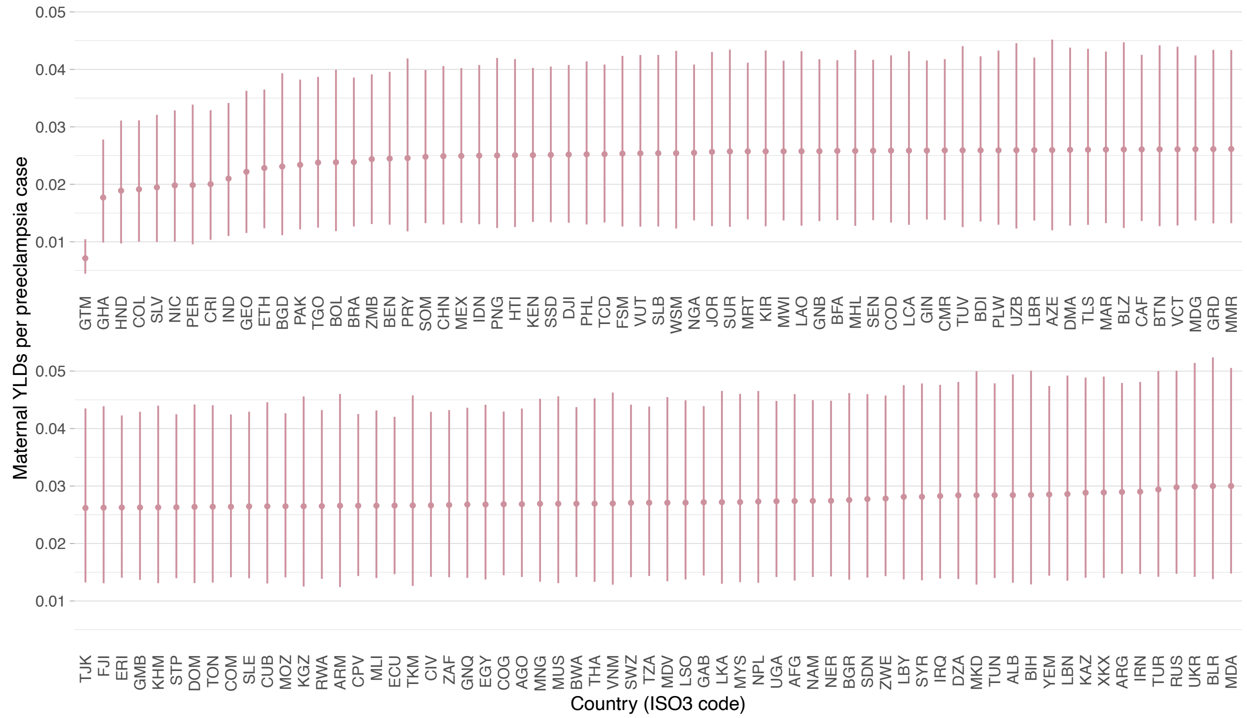

Fig C-12: Country-specific input values (maternal YLDs per preeclampsia case ($o_i^{mat\_pe}$)).

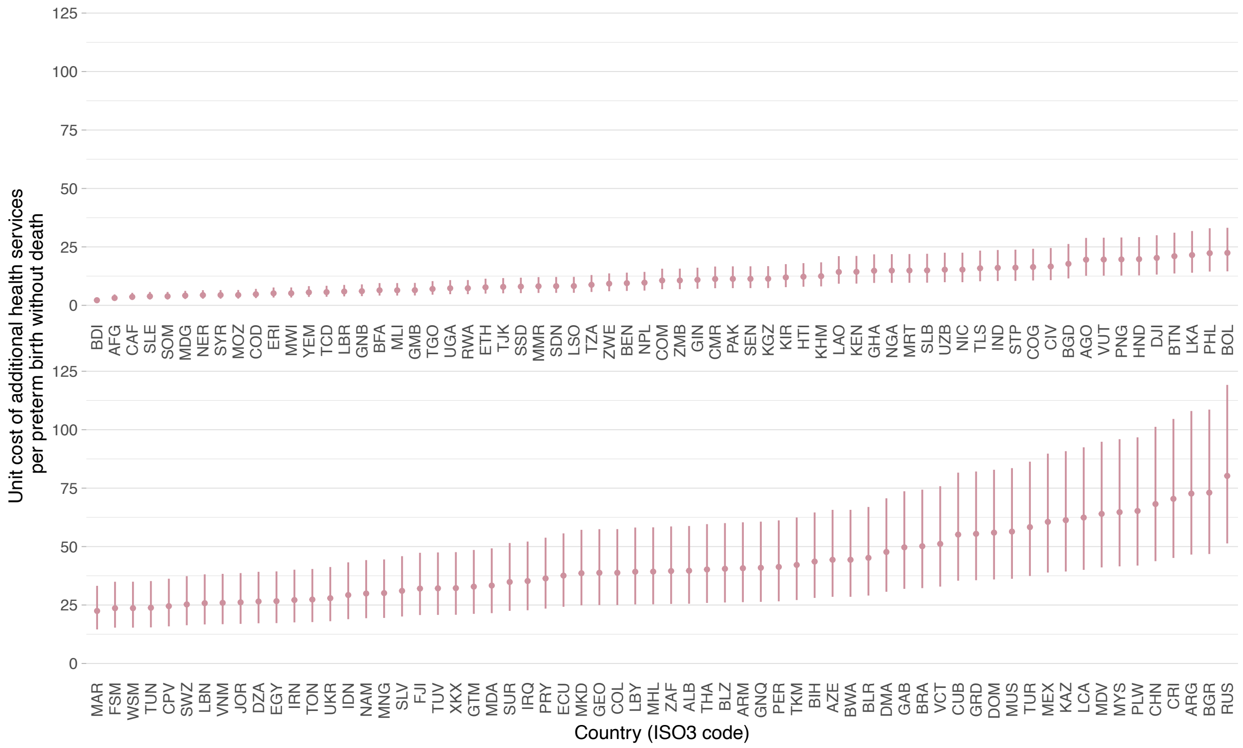

Fig C-13: Country-specific input values (unit cost for preterm birth without death (p_i^{ptb1})).

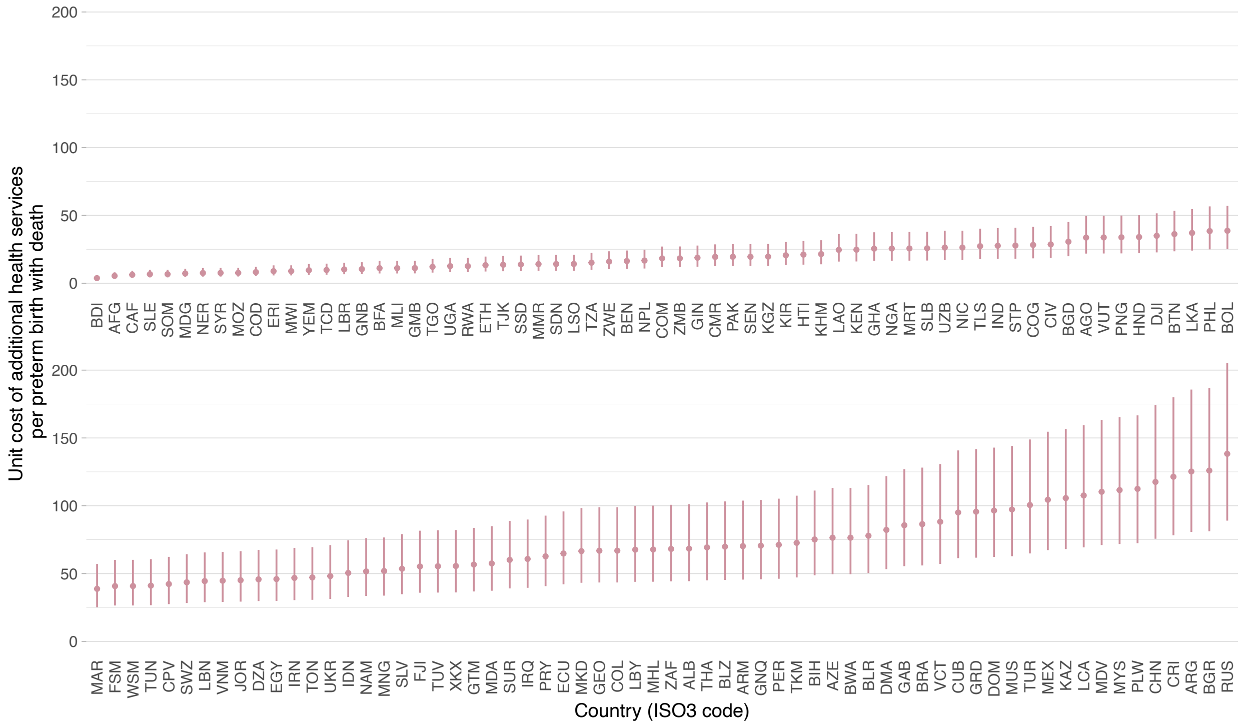

Fig C-14: Country-specific input values (unit cost for preterm birth with death (p_i^{ptb2})).

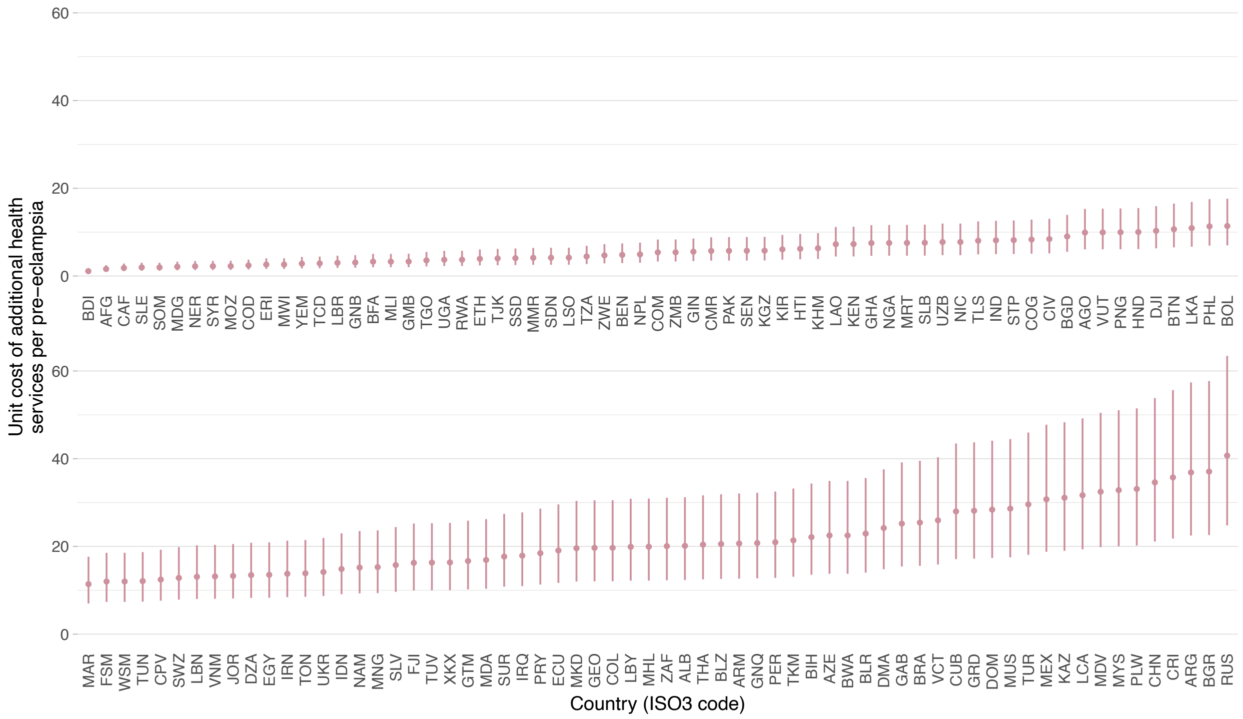

Fig C-15: Country-specific input values (unit cost for preeclampsia (p_i^{pe})).

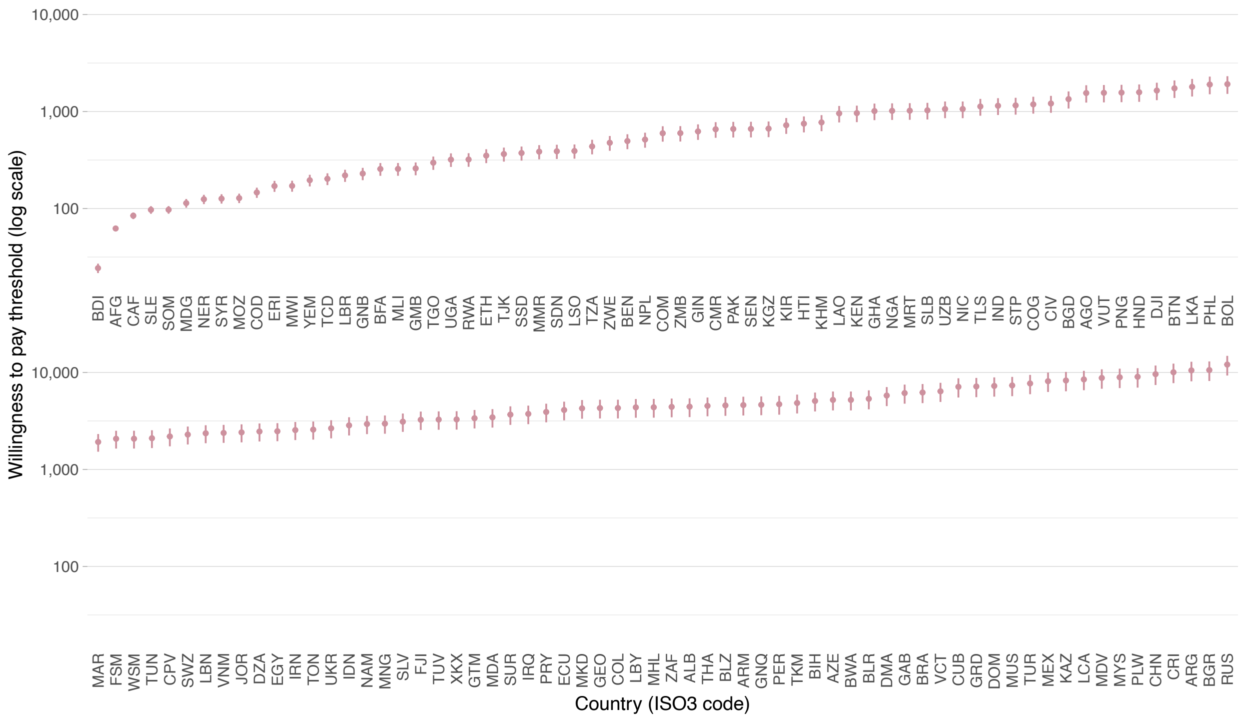

Fig C-16: Country-specific input values (willingness to pay threshold (λ_i)).

Supplementary methods

Imputation and data processing for country-level inputs

Data input	Description of data processing and imputation
Indicator for whether a country is classified as low-dietary calcium (a_i)	A binary indicator was assigned for each country to denote low dietary calcium intake (1.0 indicating low-calcium settings, 0.0 otherwise). A country was classified as having low calcium intake if at least 25% of its female population aged 15-49 years old consume <800 mg of calcium per day. Estimates of average calcium consumption were sourced from the Global Dietary Database, with estimates for countries with missing values imputed using the regional average.* The distribution of calcium consumption within each country was modeled using a gamma distribution, parameters of which were estimated via the nutriR package, utilizing regional mean intake values.[1,2] Intake data were stratified by age, and population proportions were based on United Nations demographic estimates.[4] Calculations were performed for each age cohort and aggregated to determine the national prevalence of calcium intake <800 mg per day in the age group of interest. We assumed on uncertainty in this input.
ANC coverage (b_i)	We extracted ANC coverage data for 2015 to 2021 from UNICEF reporting, operationalized as the proportion of women reporting 4+ ANC visits during their most recent pregnancy in the past 3 years.[3] To estimate mean values for ANC coverage we used the most recently reported values for each country. To quantify the uncertainty in ANC coverage we estimated the standard deviation of ANC data for each country. For countries with <3 estimates for ANC coverage reported of the 2015-2021 period, we instead estimated the standard deviation using the mean standard deviation for all countries possessing adequate records. For countries with no ANC coverage estimates over the period of interest we used the mean value and standard deviation averages across countries in the region (WHO regional grouping). For each country, we created Beta distributions matching these mean and standard deviation values.
Number of livebirths for 2024 (c_i)	We calculated mean values for this input based on point estimates reported in the UN Population Division World Population Prospects (2022 revision) for this input.[4] We calculated the uncertainty in this input for each country based on the difference between estimates from the 'low' and 'high' projection scenarios. For each country, we created Gamma distributions matching these mean and standard deviation values.
Proportion of births that are preterm (d_i)	Point estimates and uncertainty intervals for this input were extracted from a published study.[5] We used these to parameterize Beta distributions for each country.
Proportion of births with preeclampsia (e_i)	Point estimates and uncertainty intervals for the incidence of maternal hypertensive disorders were extracted from the Global Burden of Disease Study. We multiplied these values by the estimated fraction of all cases of maternal hypertensive disorders that are preeclampsia or eclampsia (37% (28, 43)),[7] and divided by the number of births for each country.[6] We used the resulting values to parameterize Beta distributions for each country.
Intervention adherence (h_i)	We extracted data describing reported consumption of iron-folate supplementation from recent (post-2015) Demographic and Health Surveys.[9] High adherence was quantified as the proportion of pregnant women who reported consuming iron-folate supplements for >90 days among women attending 4+ ANC visits at their most recent pregnancy in the last 3 years. Using these country-level values we fit a logistic regression model, with predictors including log GDP per capita and calendar year, to estimate adherence rates for each country and impute missing values. We used the results of this analysis to parameterize Beta distributions for each country.

Data input	Description of data processing and imputation
Infant deaths per preterm birth ($m_i^{inf\_ptb}$)	For each country we extracted estimates of total incidence of preterm birth and total infant deaths due to preterm birth from the Global Burden of Disease Study.[6] Infant deaths per preterm birth was calculated as the ratio of deaths to incidence. Uncertainty in these inputs was based on the uncertainty interval for deaths divided by the point estimate for incidence. For countries with missing values we used the regional mean values. We used the results of this analysis to parameterize Beta distributions for each country.
Maternal deaths per preeclampsia case ($m_i^{mat\_pe}$)	For each country we extracted estimates of total maternal deaths due to maternal hypertensive disorders from the Global Burden of Disease Study.[6] Maternal deaths per preeclampsia case birth was calculated by dividing maternal deaths by preeclampsia incidence. Uncertainty in these inputs was based on the uncertainty interval for deaths divided by the point estimate for incidence. For countries with missing values we used the regional mean values. We used the results of this analysis to parameterize Beta distributions for each country.
Infant YLLs per preterm birth ($n_i^{inf\_ptb}$)	For each country we extracted estimates of total incidence of preterm birth and total YLLs due to preterm birth from the Global Burden of Disease Study.[6] Infant YLLs per preterm birth was calculated as the ratio of total YLLs to incidence. Uncertainty in these inputs was based on the uncertainty interval for YLLs divided by the point estimate for incidence. For countries with missing values we used the regional mean values. We used the results of this analysis to parameterize Gamma distributions for each country.
Maternal YLLs per preeclampsia case ($n_i^{mat\_pe}$)	For each country we extracted estimates of total YLLs due to maternal hypertensive disorders from the Global Burden of Disease Study.[6] Maternal YLLs per preeclampsia case was calculated calculated by dividing total YLLs by preeclampsia incidence. Uncertainty in these inputs was based on the uncertainty interval for YLLs divided by the point estimate for incidence. For countries with missing values we used the regional mean values. We used the results of this analysis to parameterize Gamma distributions for each country.
Infant YLDs per preterm birth ($o_i^{inf\_ptb}$)	For each country we extracted estimates of total incidence of preterm birth and total YLDs due to preterm births by age group from the Global Burden of Disease Study.[6] Infant YLDs per preterm birth was calculated as the ratio of total YLDs incurred in the youngest age group to incidence. Uncertainty in these inputs was based on the uncertainty interval for YLDs divided by the point estimate for incidence. For countries with missing values we used the regional mean values. We used the results of this analysis to parameterize Gamma distributions for each country.
Maternal YLDs per preterm birth ($o_i^{mat\_ptb}$)	For each country we extracted estimates of total incidence of preterm birth and total YLDs due to preterm births by age group from the Global Burden of Disease Study.[6] Maternal YLDs per preterm birth was calculated as the ratio of total YLDs incurred outside of the youngest age group to incidence. Uncertainty in these inputs was based on the uncertainty interval for YLDs divided by the point estimate for incidence. For countries with missing values we used the regional mean values. We used the results of this analysis to parameterize Gamma distributions for each country.
Maternal YLDs per preeclampsia case ($o_i^{mat\_pe}$)	For each country we extracted estimates of total incidence of maternal hypertensive disorders and total YLDs due to maternal hypertensive disorders from the Global Burden of Disease Study.[6] Maternal YLDs per preeclampsia case was calculated by dividing total YLDs by preeclampsia incidence. Uncertainty in these inputs was based on the uncertainty interval for YLDs divided by the point estimate for incidence. For countries with missing values we used the regional mean values. We used the results of this analysis to parameterize Gamma distributions for each country.

Data input	Description of data processing and imputation
Unit cost of additional health services per preterm birth without death (p_i^{ptb1})	The unit cost of additional health services per preterm birth without death was calculated from a linear regression model fit to published data for 6 countries.[10-12] The model utilized the log unit cost as the dependent variable, with the birth outcome (costs with preterm birth without deaths versus term birth) and country log GDP per capita as independent variables. We used this model to estimate unit costs for each country.
Unit cost of additional health services per preterm birth with death (p_i^{ptb2})	Similar to p_i^{ptb1}, the unit cost of additional health services per preterm birth with death was calculated from a linear regression model fit to published data for 6 countries.[10-12] The model utilized the log unit cost as the dependent variable, with the birth outcome (costs with preterm birth with death versus term birth) and country log GDP per capita as independent variables. We used this model to estimate unit costs for each country.
Unit cost of additional health services per pre-eclampsia episode (p_i^{pe})	Similar to p_i^{ptb1}, the unit cost of additional health services per pre-eclampsia episode was calculated from a linear regression model fit to published data for 5 countries.[10,12] The model utilized the log unit cost as the dependent variable, with the birth outcome (costs with high-risk vs. low-risk pregnancy) and country log GDP per capita as independent variables. We used this model to estimate unit costs for each country.
Willingness to pay (WTP) threshold (λ_i)	For each country, we extracted estimates of the range of WTP values from a published source, operationalized as the WTP threshold as a percentage of per capita GDP.[16] We used the mid-point of these values as the mean, and the range to create an interval. We imputed missing values using a linear regression model, using log GDP per capita as a predictor. For each country, we multiplied these values by current per capita GDP to obtain the WTP threshold. We used the results of this analysis to parameterize Uniform distributions for each country.

Table H: Methods used for imputation and data processing for country-level inputs.

* The availability of input values for each country is shown in Figure S1.

Equations used to calculate each study outcome.

For each country, the number of cases of preterm birth averted (k_i):

$$k_i = a_i * b_i * c_i * d_i * (1 - f) * (h_i + (1 - h_i) * j)$$

For each country, the number of cases of preeclampsia averted (l_i):

$$l_i = a_i * b_i * c_i * e_i * (1 - g) * (h_i + (1 - h_i) * j)$$

For each country, the number of infant and maternal deaths averted ($deaths_i^{inf}$, $deaths_i^{mat}$):

$$deaths_i^{inf} = k_i * m_i^{inf\_ptb}$$

$$deaths_i^{mat} = l_i * m_i^{mat\_pe}$$

For each country, infant and maternal YLLs averted (YLL_i^{inf}, YLL_i^{mat}):

$$YLL_i^{inf} = k_i * n_i^{inf\_ptb}$$

$$YLL_i^{mat} = l_i * n_i^{mat\_pe}$$

For each country, infant and maternal YLDs averted (YLD_i^{inf}, YLD_i^{mat}):

$$YLD_i^{inf} = k_i * o_i^{inf\_ptb}$$

$$YLD_i^{mat} = k_i * o_i^{mat\_ptb} + l_i * o_i^{mat\_pe}$$

For each country, DALYs due to preterm birth averted ($DALY_i^{ptb}$):

$$DALY_i^{ptb} = k_i * \left(n_i^{inf\_ptb} + o_i^{inf\_ptb} + o_i^{mat\_ptb}\right)$$

For each country, DALYs due to preeclampsia averted ($DALY_i^{pe}$):

$$DALY_i^{pe} = l_i * \left(n_i^{mat\_pe} + o_i^{mat\_pe}\right)$$

For each country, DALYs due to premature death averted (YLL_i^{total}):

$$YLL_i^{total} = YLL_i^{inf} + YLL_i^{mat}$$

For each country, DALYs due to non-fatal health losses averted (YLD_i^{total})

$$YLD_i^{total} = YLD_i^{inf} + YLD_i^{mat}$$

For each country, infant, maternal, and total DALYs averted ($DALY_i^{inf}$, $DALY_i^{mat}$, $DALY_i^{total}$):

$$DALY_i^{inf} = YLL_i^{inf} + YLD_i^{inf}$$

$$DALY_i^{mat} = YLL_i^{mat} + YLD_i^{mat}$$

$$DALY_i^{total} = DALY_i^{inf} + DALY_i^{mat}$$

For each country, intervention costs ($Cost_i^{calcium}$):

$$Cost_i^{calcium} = a_i * b_i * c_i * p^{calc} * 140 * (1 + q^{sc})/(1 - q^{waste})$$

For each country, cost savings from averted preterm births and averted preeclampsia cases ($Cost_i^{avert\_ptb}$, $Cost_i^{avert\_ptb}$):

$$Cost_i^{avert\_ptb} = k_i * \left(p_i^{ptb1} * \left(1 - m_i^{inf\_ptb}\right) + p_i^{ptb2} * m_i^{inf\_ptb}\right)$$

$$Cost_i^{avert\_ptb} = l_i * p_i^{pe}$$

For each country, total incremental costs ($Cost_i^{total}$):

$$Cost_i^{total} = Cost_i^{calcium} - Cost_i^{avert\_ptb} - Cost_i^{avert\_pe}$$

For each country, the incremental cost per adverse pregnancy outcome averted ($ICER_i^{adv}$):

$$ICER_i^{adv} = Cost_i^{total}/(k_i + l_i)$$

For each country, the incremental cost per adverse pregnancy outcome averted ($ICER_i^{DALY}$):

$$ICER_i^{DALY} = Cost_i^{total}/DALY_i^{total}$$

For each country, the net monetary benefit (NMB_i):

$$NMB_i = DALY_i^{total} * \lambda_i - Cost_i^{total}$$

For each country, the return on investment (ROI_i:

$$ROI_i = \left(DALY_i^{total} * \lambda_i - Cost_i^{total}\right)/Cost_i^{calcium}$$

For each country, the cost-savings versus. an intervention requiring 1500mg calcium per day (2 additional pills per day) (CS_i):

$$CS_i = Cost_i^{calcium} * \frac{420 - 140}{140}$$

Citations

1. Passarelli S, Free CM, Allen LH, et al. Estimating national and subnational nutrient intake distributions of global diets. *The American Journal of Clinical Nutrition* 2022; **116**(2): 551-60.

2. Global Dietary Database. Global Dietary Database 2018 Estimates: Dietary Calcium [https://www.globaldietarydatabase.org/data-download]. Boston, USA: Global Dietary Database, 2021.

3. WHO Global Health Observatory. Indicator Metadata Registry List: Antenatal care coverage - at least four visits (%) [https://www.who.int/data/gho/indicator-metadata-registry/imr-details/80]. Geneva: World Health Organization, 2023.

4. United Nations Population Division. World Population Prospects 2022, Online Edition [https://population.un.org/wpp/]. Geneva: United Nations, Department of Economic and Social Affairs, 2022.

5. Ohuma EO, Moller A-B, Bradley E, et al. National, regional, and global estimates of preterm birth in 2020, with trends from 2010: a systematic analysis. *The Lancet* 2023; **402**(10409): 1261-71.

6. Global Burden of Disease Collaborative Network. Global Burden of Disease Study 2019 (GBD 2019) Results [https://vizhub.healthdata.org/gbd-results/]. Seattle, USA: Institute for Health Metrics and Evaluation (IHME), 2020.

7. Magee LA, Sharma S, Nathan HL, et al. The incidence of pregnancy hypertension in India, Pakistan, Mozambique, and Nigeria: A prospective population-level analysis. *PLoS Med* 2019; **16**(4): e1002783.

8. Hofmeyr GJ, Lawrie TA, Atallah ÁN, Torloni MR. Calcium supplementation during pregnancy for preventing hypertensive disorders and related problems. *Cochrane database of systematic reviews* 2018; (10).

9. ICF. Demographic and Health Surveys (various) 2004-2022 [https://dhsprogram.com/data/available-datasets.cfm]. Rockville, Maryland: ICF 2023.

10. Bresnahan B, Vodicka E, Babigumira J, et al. Cost estimation alongside a multi-regional, multi-country randomized trial of antenatal ultrasound in five low-and-middle-income countries. *BMC Public Health* 2021; **21**(1): 952.

11. Patterson JK, Neuwahl S, Goco N, et al. Cost-effectiveness of low-dose aspirin for the prevention of preterm birth: a prospective study of the Global Network for Women's and Children's Health Research. *The Lancet Global Health* 2023; **11**(3): e436-e44.

12. Stenberg K, Lauer JA, Gkountouras G, Fitzpatrick C, Stanciole A. Econometric estimation of WHO-CHOICE country-specific costs for inpatient and outpatient health service delivery. *Cost Effectiveness and Resource Allocation* 2018; **16**(1): 11.

13. World Health Organization. WHO antenatal care recommendations for a positive pregnancy experience: nutritional interventions update: multiple micronutrient supplements during pregnancy. 2020.

14. Bertram MY, Stenberg K, Brindley C, et al. Disease control programme support costs: an update of WHO-CHOICE methodology, price databases and quantity assumptions. *Cost Eff Resour Alloc* 2017; **15**: 21.

15. Guadie M, Asemahagn MA, Tefera A, Melkam W, Habteweld HA, Derebe D. Medicines Wastage and Its Contributing Factors in Public Health Facilities of South Gondar Zone, Amhara Regional State, Ethiopia. *Integrated Pharmacy Research and Practice* 2023: 157-70.

16. Ochalek J, Lomas J, Claxton K. Estimating health opportunity costs in low-income and middle-income countries: a novel approach and evidence from cross-country data. *BMJ Global Health* 2018; **3**: e000964.
